# Supplementary material for: Simultaneous evolutionary expansion and constraint of genomic heterogeneity in multifocal lung cancer
Source: Nat Commun. 2017 Oct 10;8:823. doi: 10.1038/s41467-017-00963-0 (PMC5634994; doi:10.1038/s41467-017-00963-0)
Supplement: Supplementary file 1 — Supplementary Information [file 41467_2017_963_MOESM1_ESM.pdf]

Description of Supplementary Files

File Name: Supplementary Information
Description: Supplementary Figures and Supplementary Tables

File Name: Supplementary Data 1
Description: Identification of 167-679 somatic alterations per tumor, including a total of 373 non-synonymous exonic mutations present in at least one tumor region.

File Name: Peer Review File

Supplementary Figure 1.

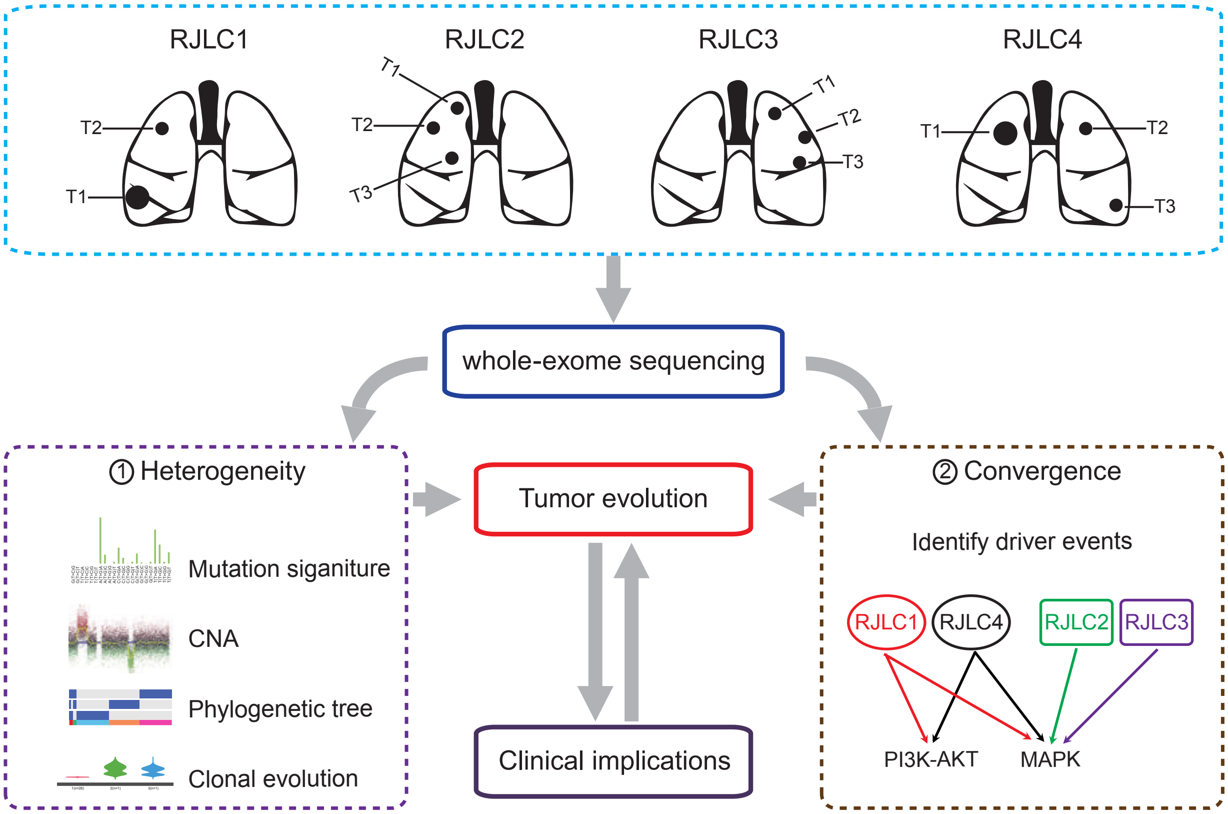

Supplementary Figure 1. A schematic overview of the study design.

Supplementary Figure 2.

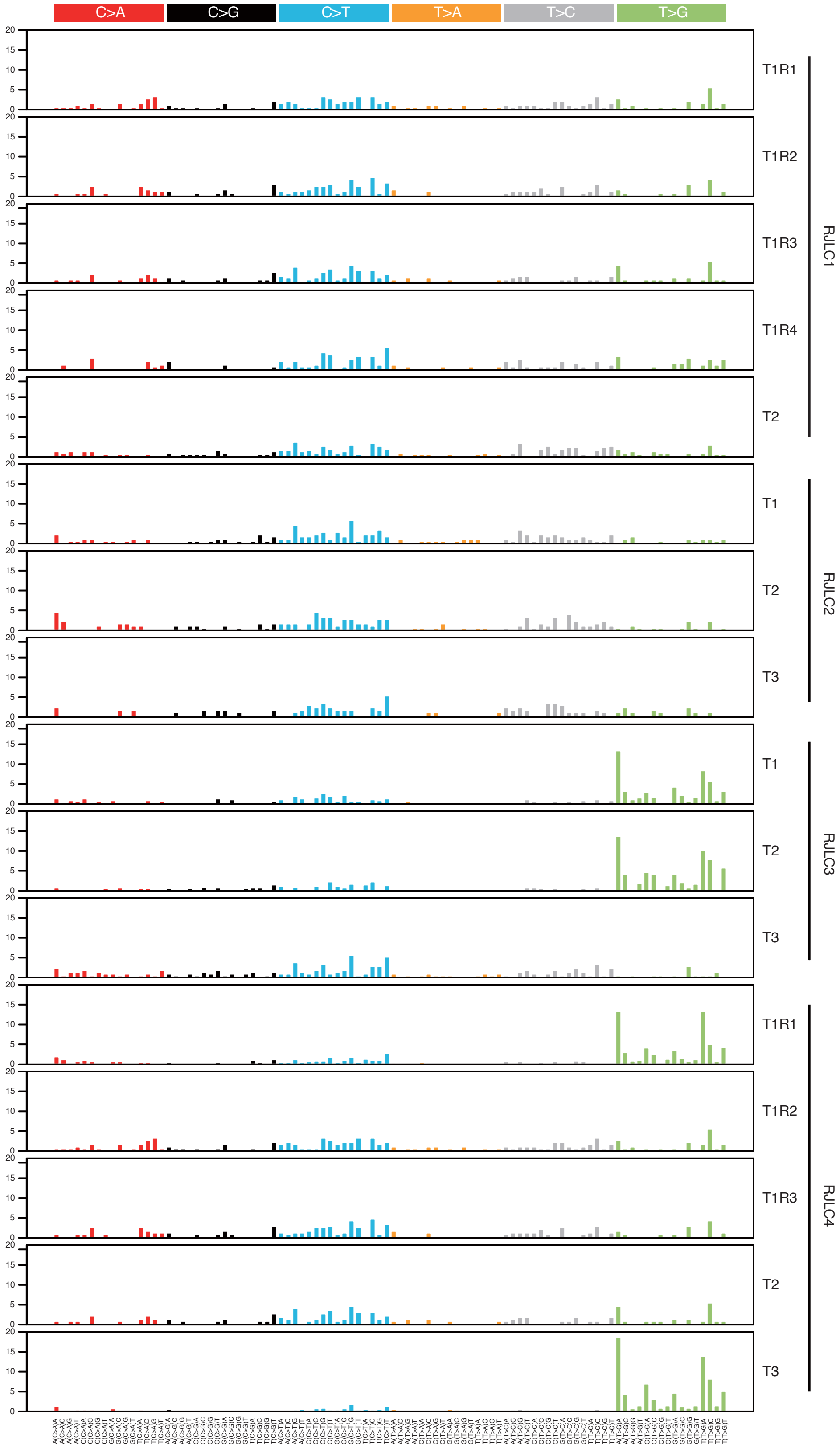

Supplementary Figure 2. Mutational signatures of sequenced tumor samples.

Supplementary Figure 3.

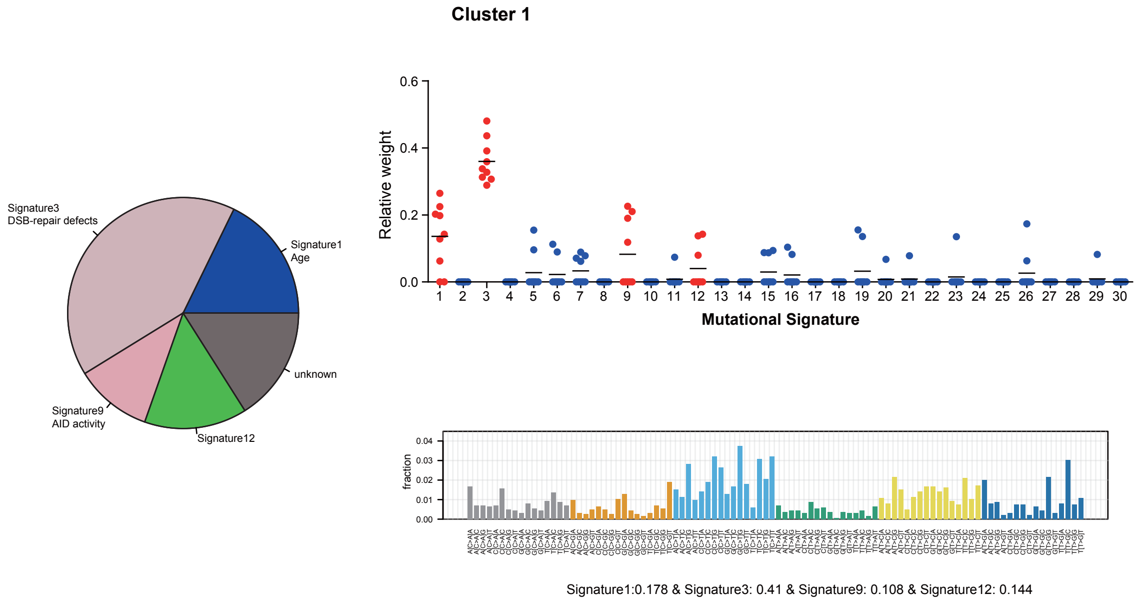

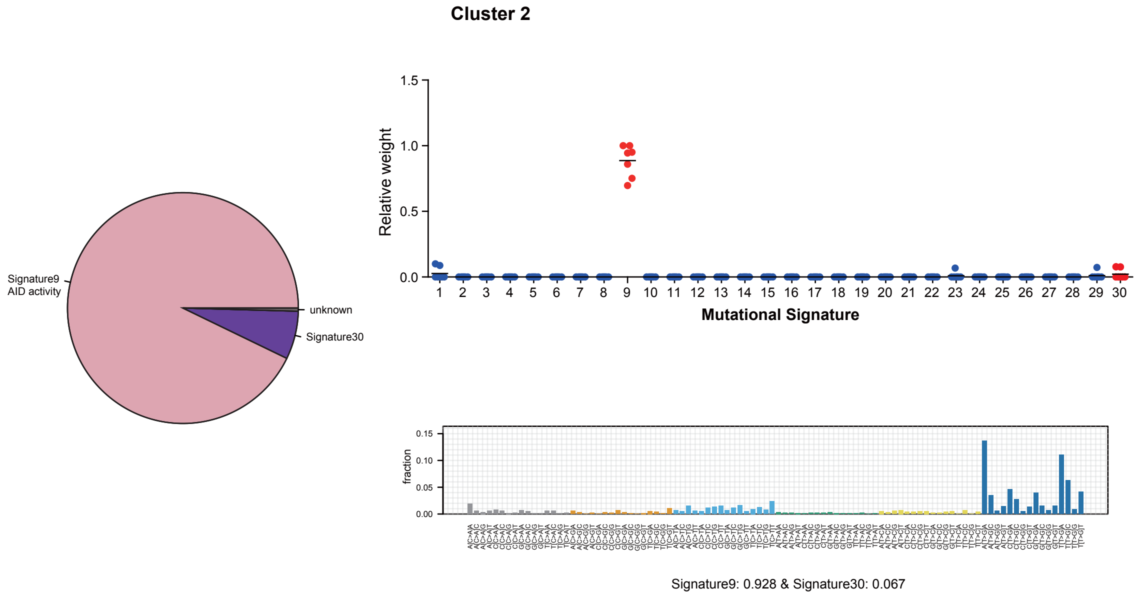

Supplementary Figure 3. A multiple regression approach, deconstructSigs, was used to extract known signatures based on the Wellcome Trust Sanger Institute Mutational Signature Framework and to statistically quantify the contribution of each signature for each cluster.

Supplementary Figure 4.

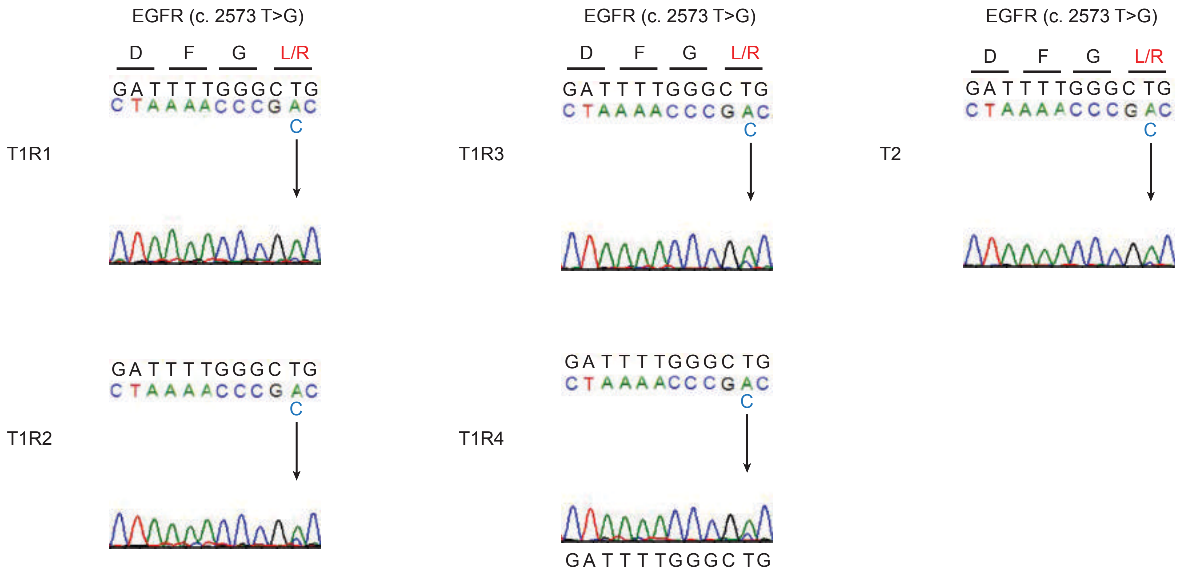

Supplementary Figure 4. Sanger sequencing validation of EGFR mutation in RJLC1.

Supplementary Figure 5.

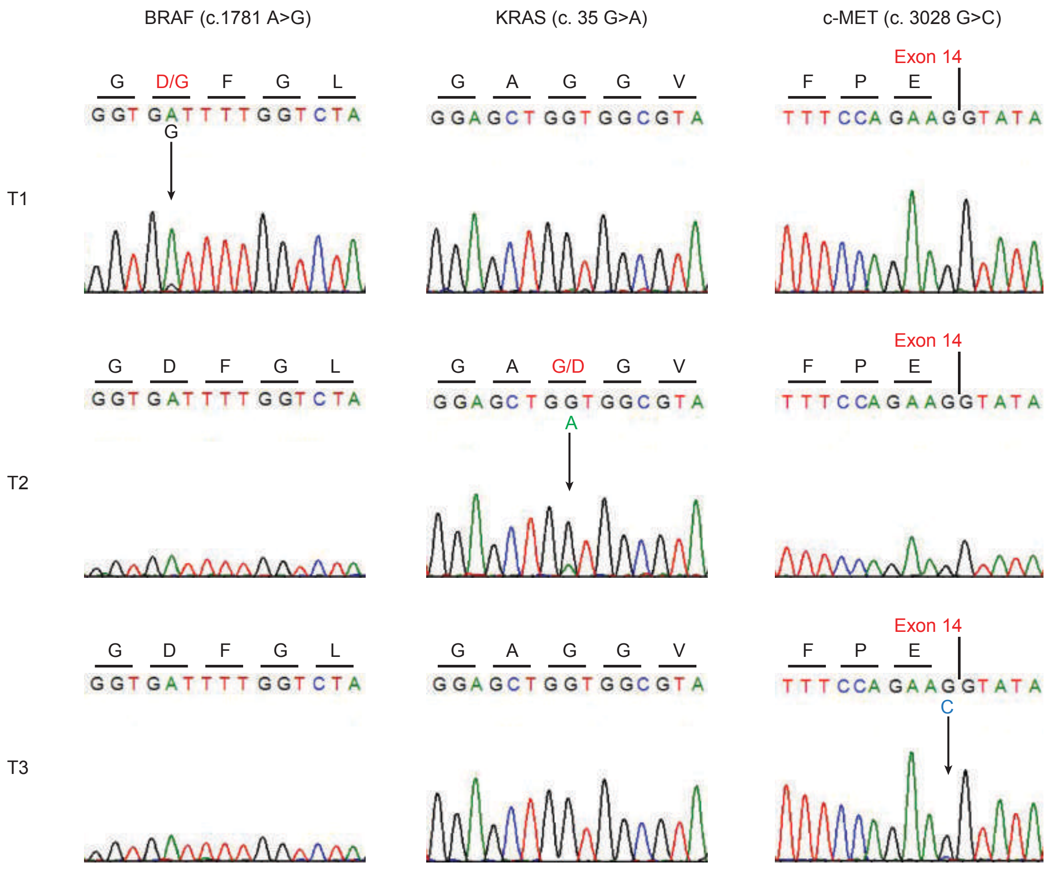

Supplementary Figure 5. Sanger sequencing validation of BRAF, KRAS and c-MET mutations in RJLC2.

Supplementary Figure 6.

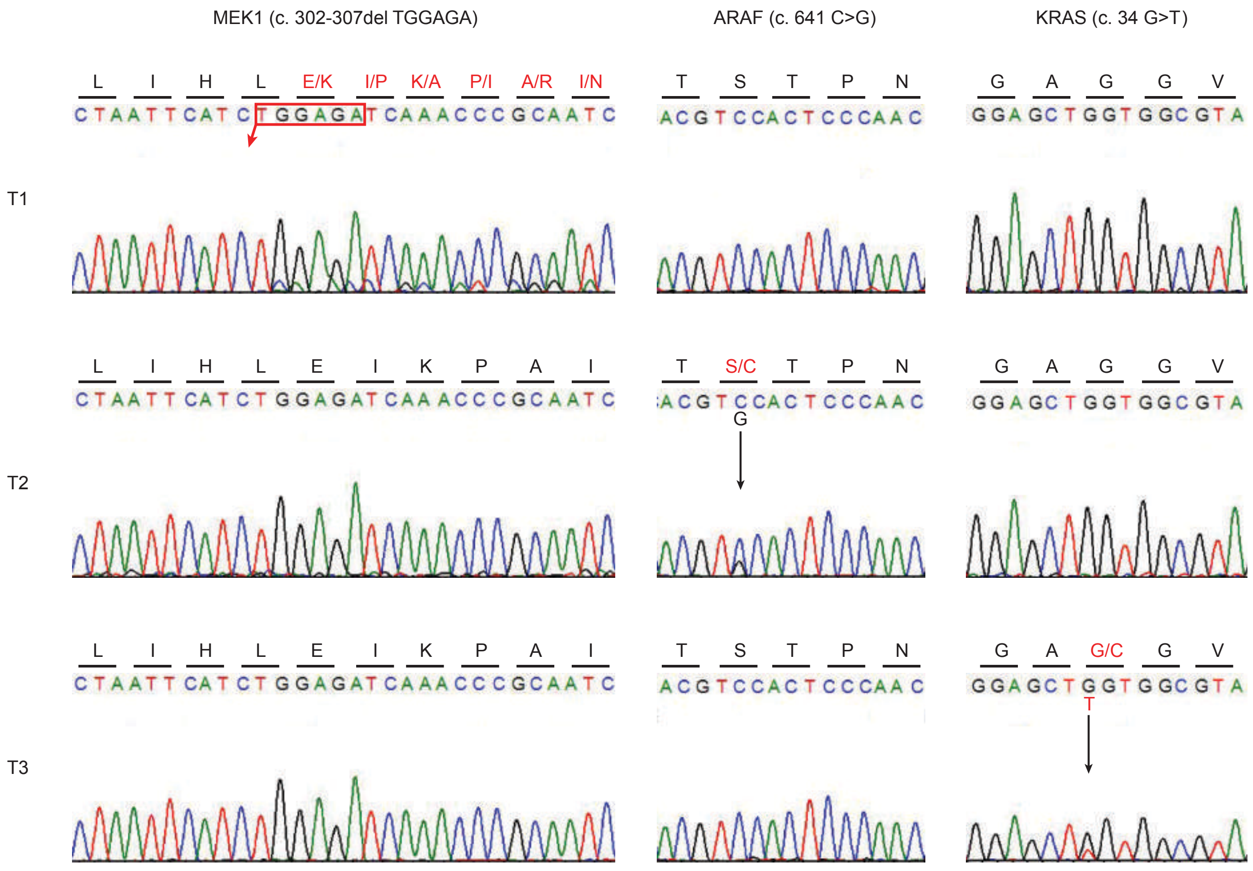

Supplementary Figure 6. Sanger sequencing validation of MEK1, ARAF and KRAS mutations in RJLC3.

Supplementary Figure 7.

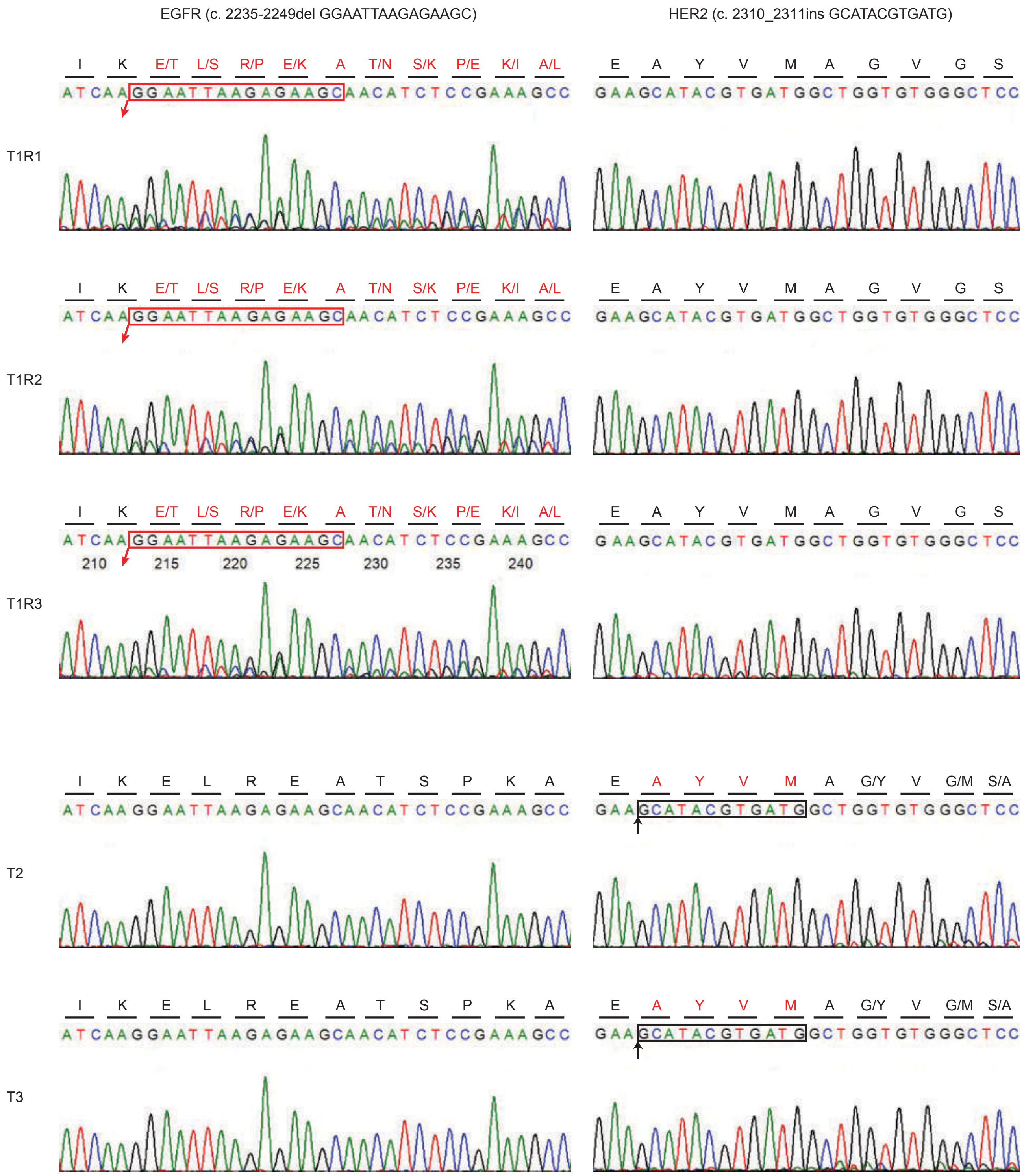

Supplementary Figure 7. Sanger sequencing validation of EGFR and HER2 mutations in RJLC4.

Supplementary Figure 8.

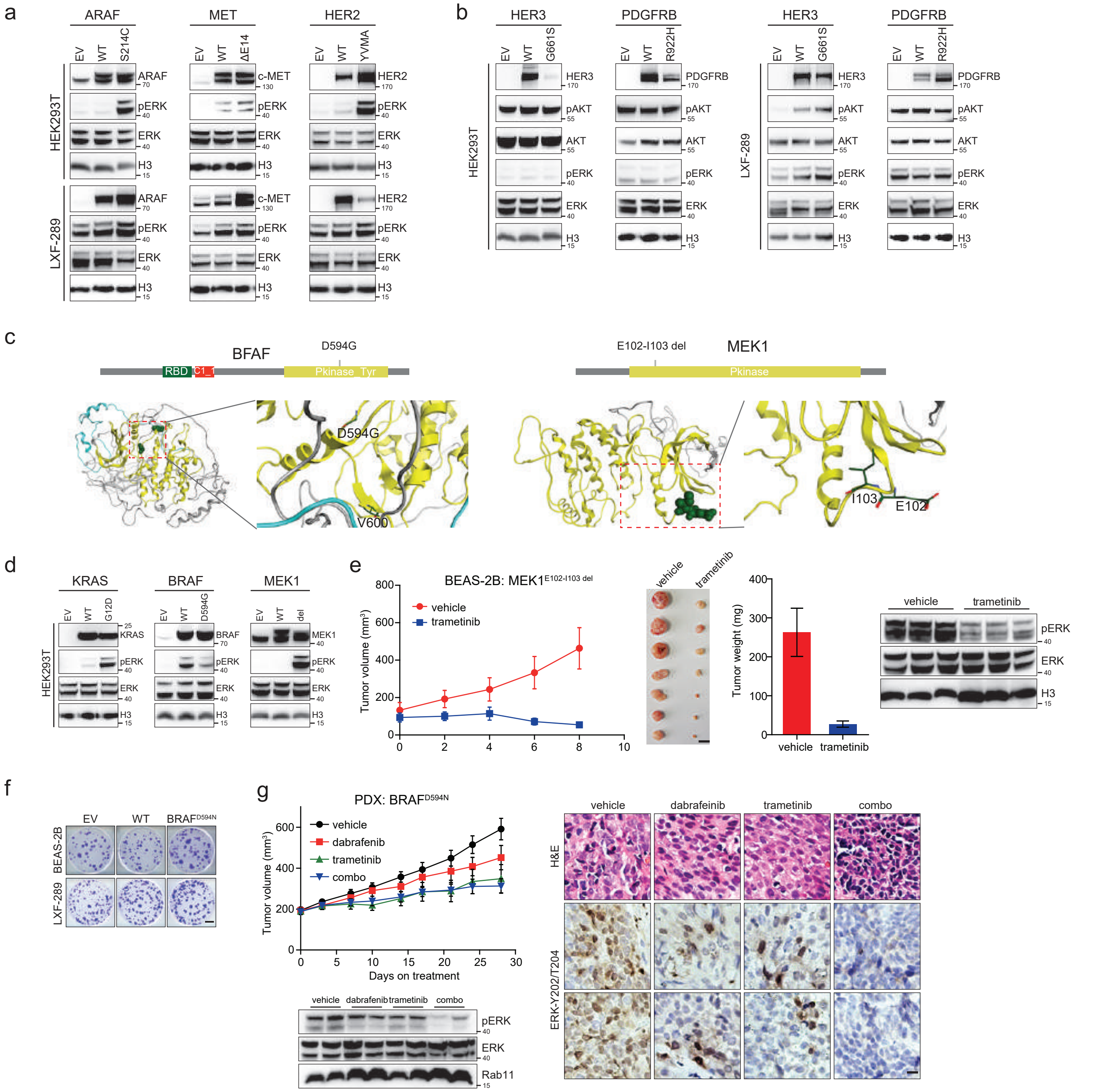

Supplementary Figure 8. (a) Overexpression of indicated wild-type or mutant genes in HEK293T or LXF-289 cells. Gene expression and ERK phosphorylation were measured by immunoblotting. (b) Overexpression of indicated wild-type or mutant genes in HEK293T or LXF-289 cells. AKT and ERK phosphorylation was measured by immunoblotting. (c) The mutated protein structures were generated by Phyre2 web portal and figures were prepared by using PyMOL. (d) Overexpression of indicated wild-type or mutant genes in HEK293T cells. Gene expression and ERK phosphorylation were measured by immunoblotting. (e) Tumor growth of BEAS-2B xenografts treated with trametinib or vehicle control. Each line represented mean tumor volume of the respective group, and error bars indicated standard deviation (10 mice per group). ERK phosphorylation was measured by immunoblotting. Scale bar, 10 mm. (f) Indicated wild-type or mutant genes were lentivirally introduced into BEAS-2B or LXF-289 cells. Cells were cultured for two weeks and stained with crystal violet. Scale bar, 5 mm. (g) Tumor growth of BRAF^{D594N} PDX treated with indicated drugs. Each line represented mean tumor volume of the respective group, and error bars indicated standard deviation (10 mice per group). ERK phosphorylation was measured by immunoblotting or IHC. Scale bar, 5 μm.

Supplementary Figure 9.

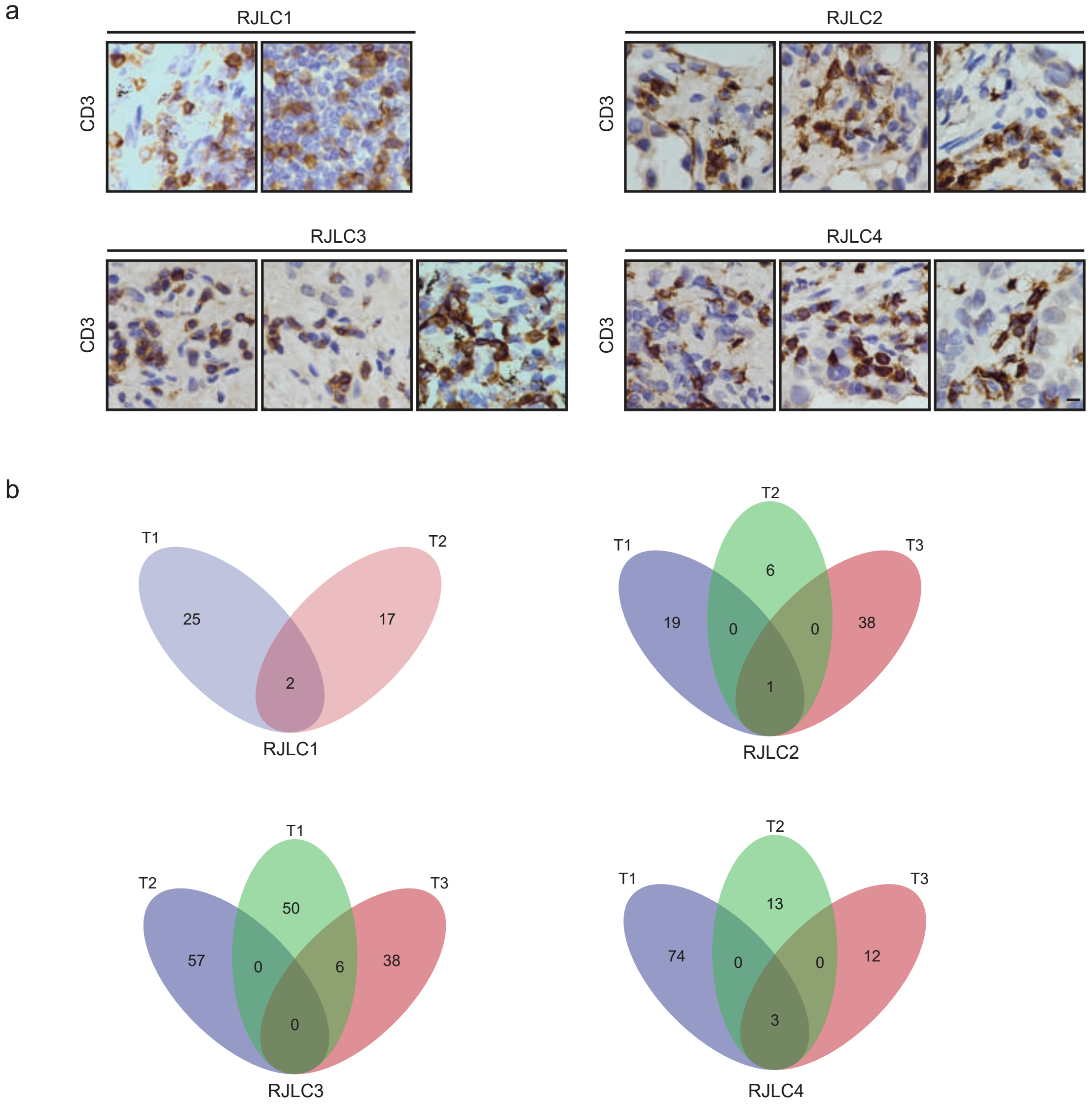

Supplementary Figure 9. (a) IHC of CD3 in different MSLC tumors. Scale bar, 5 μm. (b) Load and overlap of predicted neoantigens in each MSLC case using NetMHCpan algorithm.

Supplementary Figure 10.

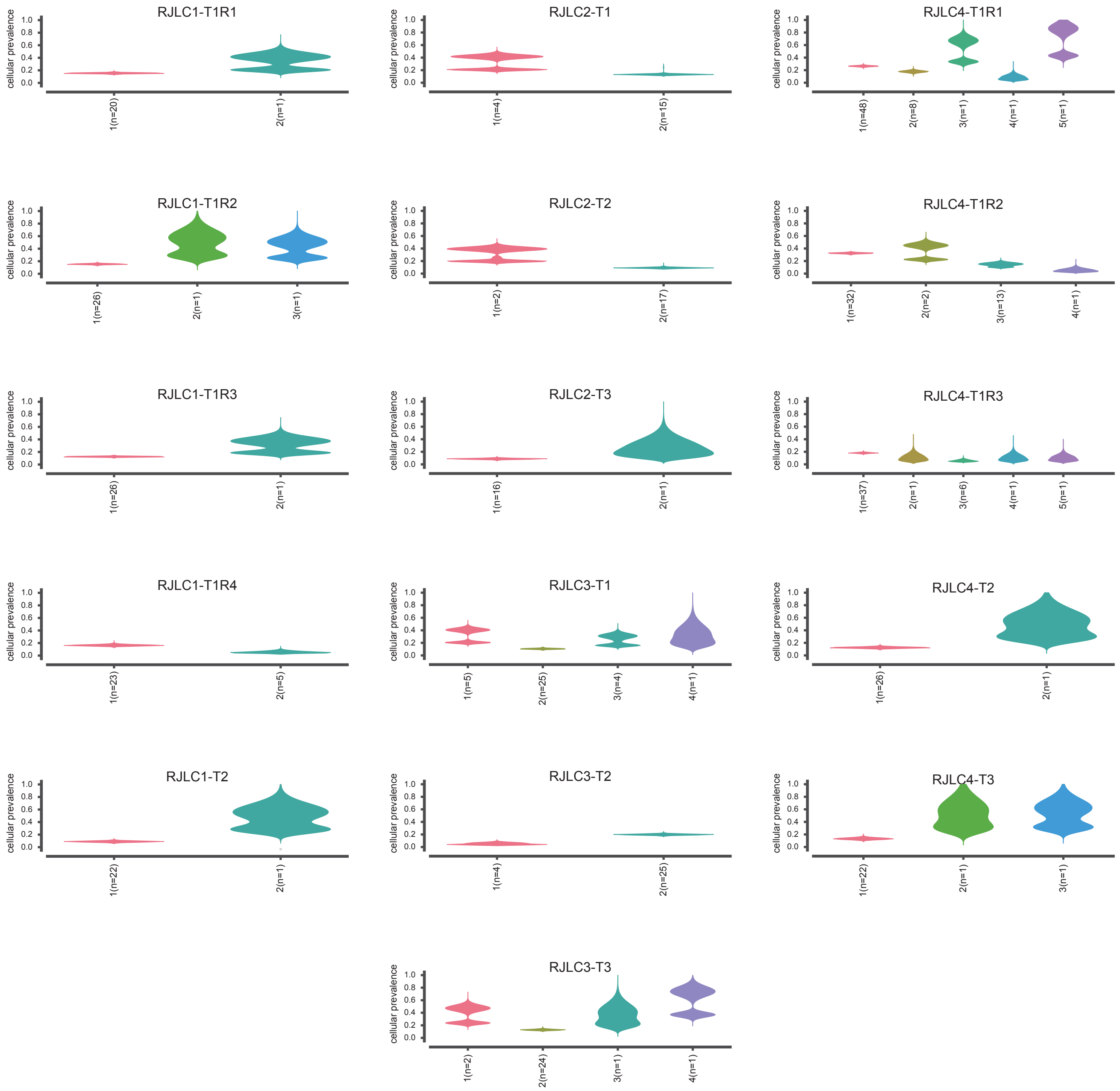

Supplementary Figure 10. PyClone analysis of sequenced tumor samples.

Supplementary Figure 11.

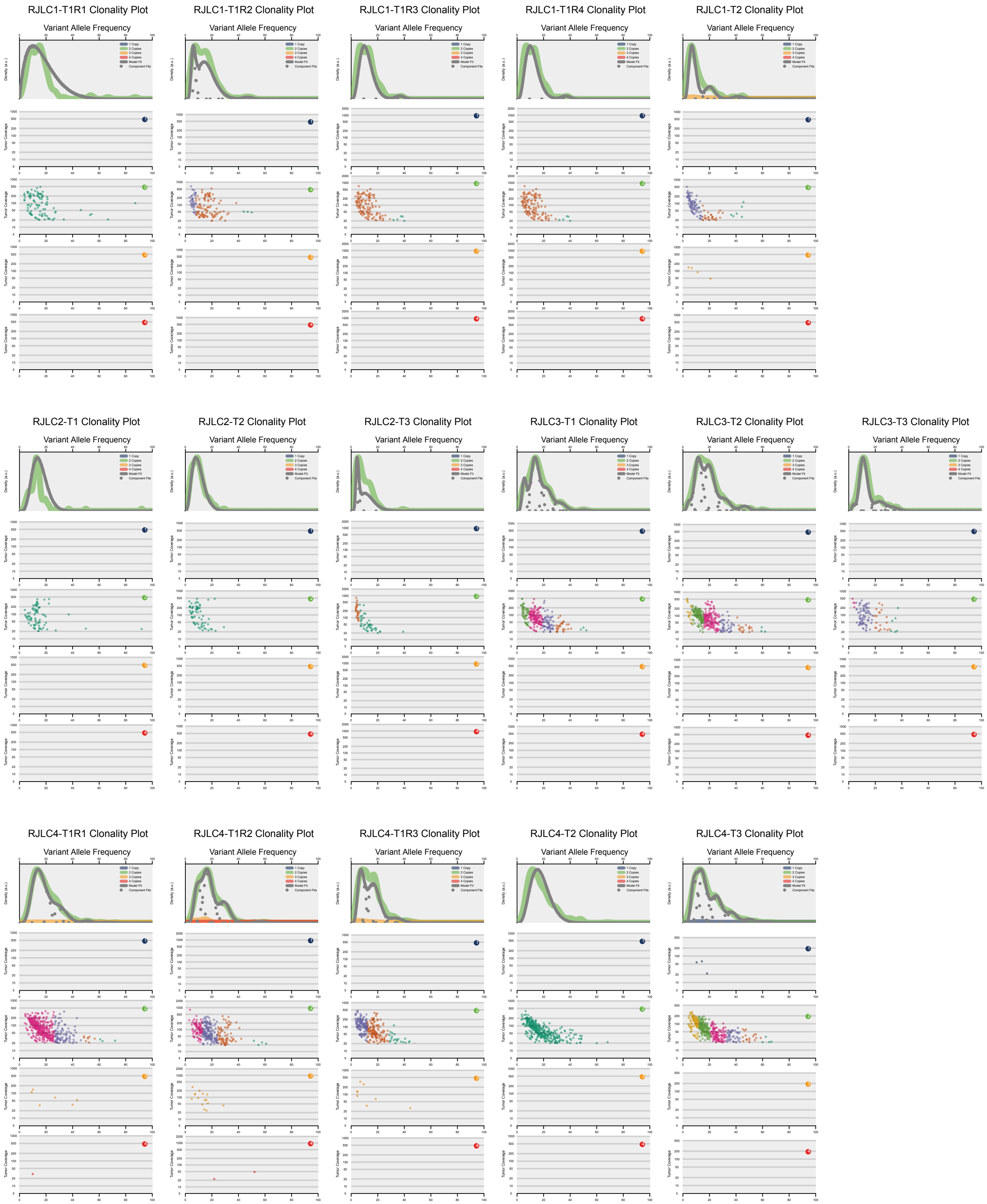

Supplementary Figure 11. SciClone analysis of sequenced tumor samples.

Supplementary Figure 12.

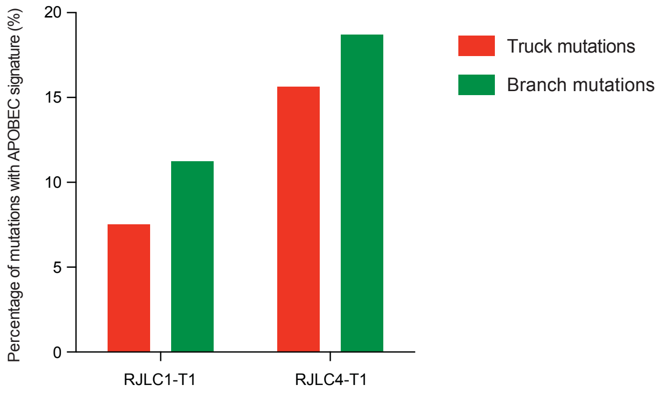

Supplementary Figure 12. Percentage of mutations with APOBEC signature for trunk and branch mutations in two M-seq tumors.

Supplementary Figure 13.

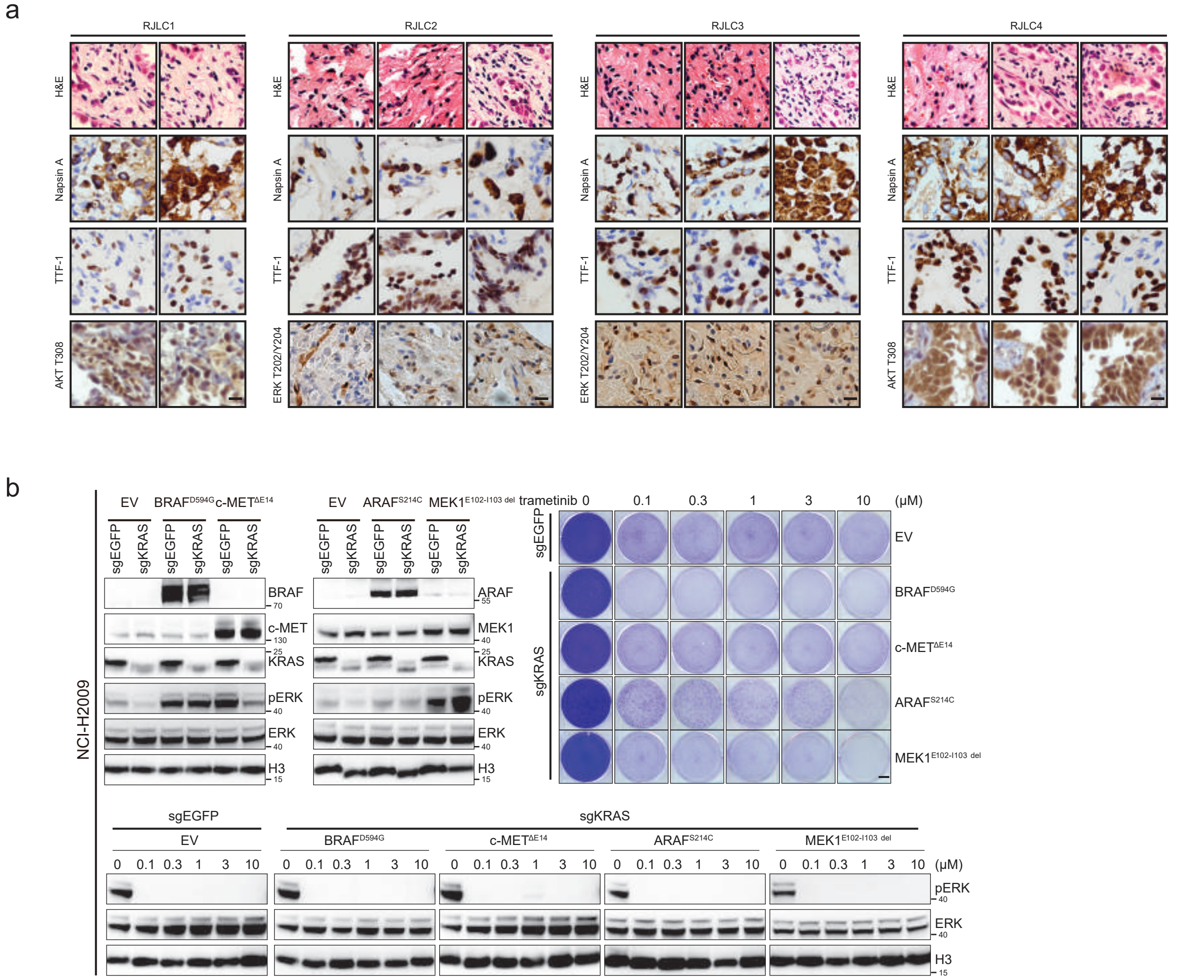

Supplementary Figure 13. (a) H&E and IHC of indicated markers in different MSLC tumors. Scale bar, 5 μm. (b) KRAS was knocked out using CRISPR-Cas9 system in NCI-H2009 cells and replaced with $BRAF^{D594G}$, $c\text{-}MET^{\Delta E14}$, $ARAF^{S214C}$ or $MEK1^{E102\text{-}I103\ del}$. ERK phosphorylation was measured by Western blot analysis. Indicated cells were treated with a serial dilution of trametinib for a week and stained with crystal violet. Scale bar, 5 mm. The corresponding cell lysates were analyzed by immunoblotting.

Supplementary Figure 14.

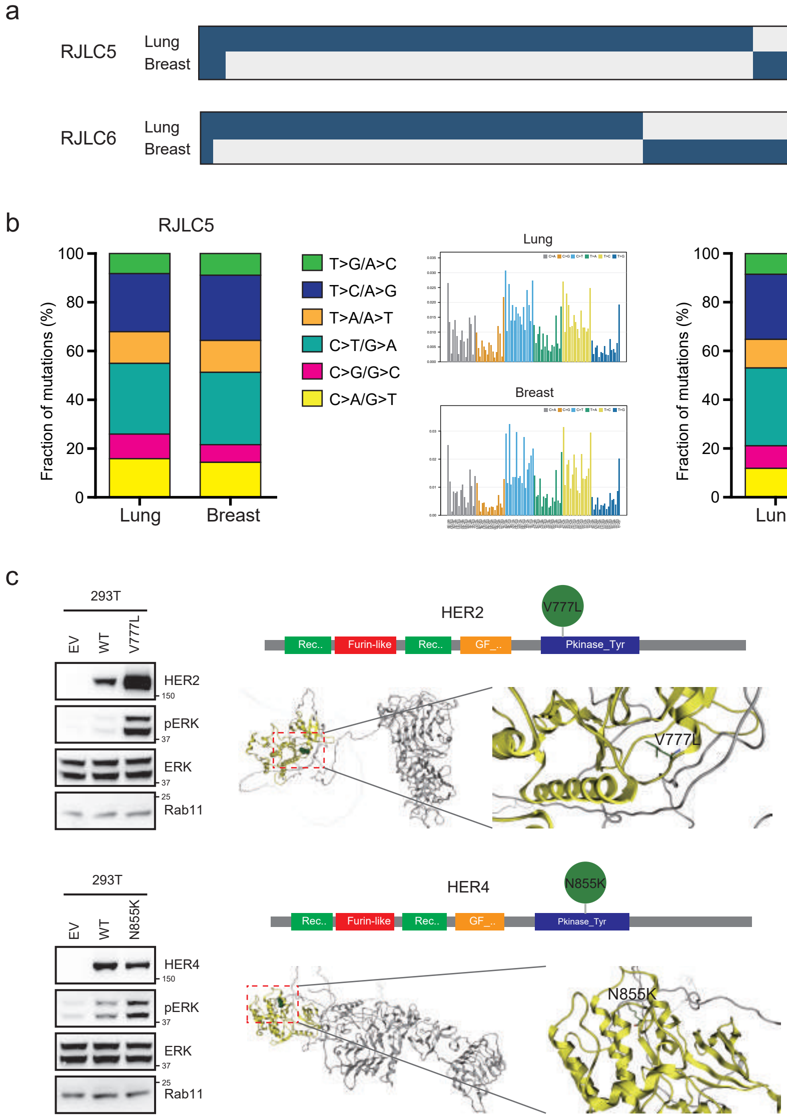

Supplementary Figure 14. (a) Heat maps showed the presence (blue) or absence (gray) of all non-silent somatic mutations in lung and breast tumors. (b) Mutation spectra and mutational signatures of lung and breast tumors in RJLC5 and RJLC6. (c) Overexpression of indicated wild-type or mutant genes in HEK293T cells. Gene expression and ERK phosphorylation were measured by immunoblotting. The mutated protein structures were generated by Phyre2 web portal and figures were prepared by using PyMOL.

Supplementary Figure 15.

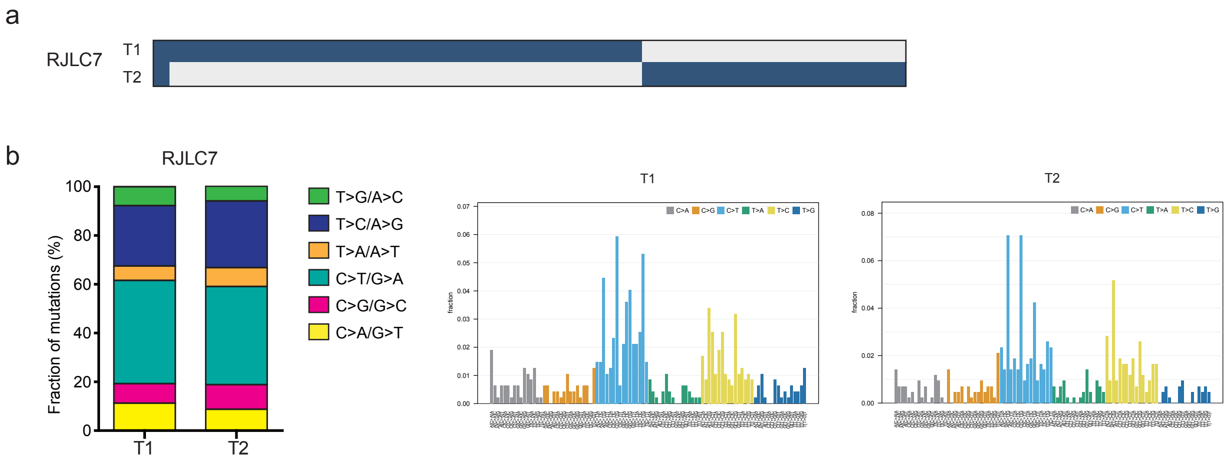

Supplementary Figure 15. (a) Heat maps showed the presence (blue) or absence (gray) of all non-silent somatic mutations in two tumors of RJLC7. (b) Mutation spectra and mutational signatures of two tumors in RJLC7.

Supplementary Figure 16.

Figure 2c

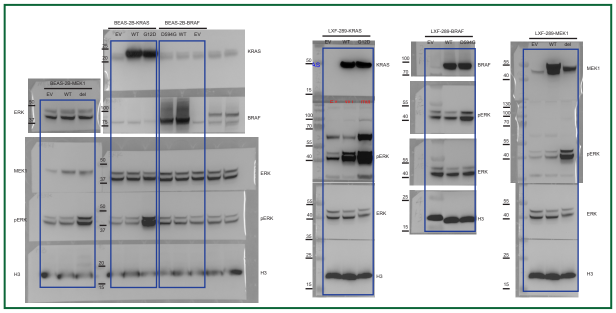

Figure 3d

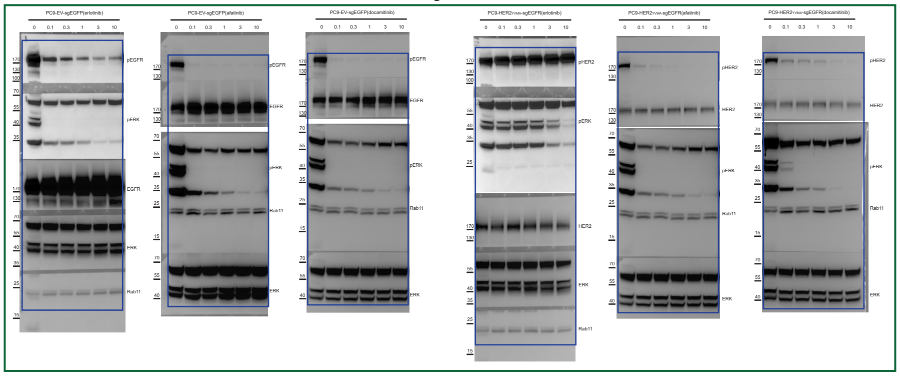

Figure 3c

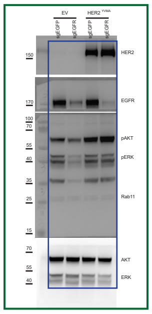

Figure 3e

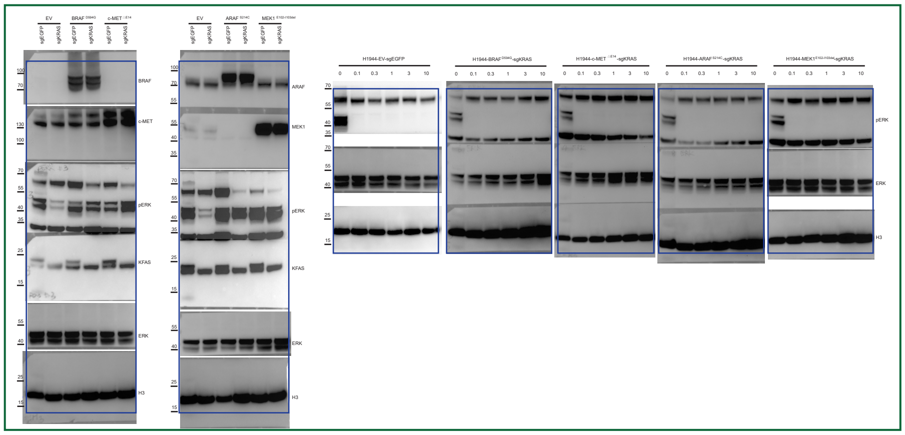

Supplementary Figure 8a

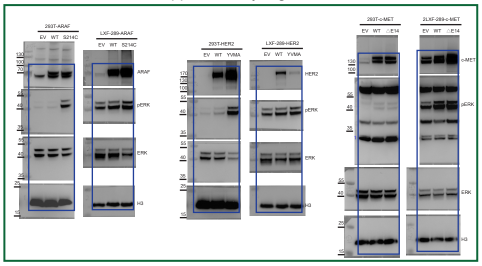

Supplementary Figure 8b

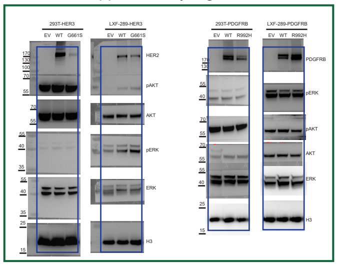

Supplementary Figure 8d

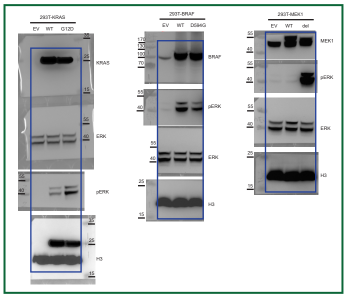

Supplementary Figure 8e

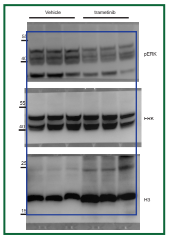

Supplementary Figure 8g

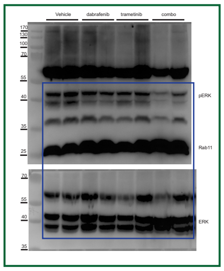

Supplementary Figure 13b

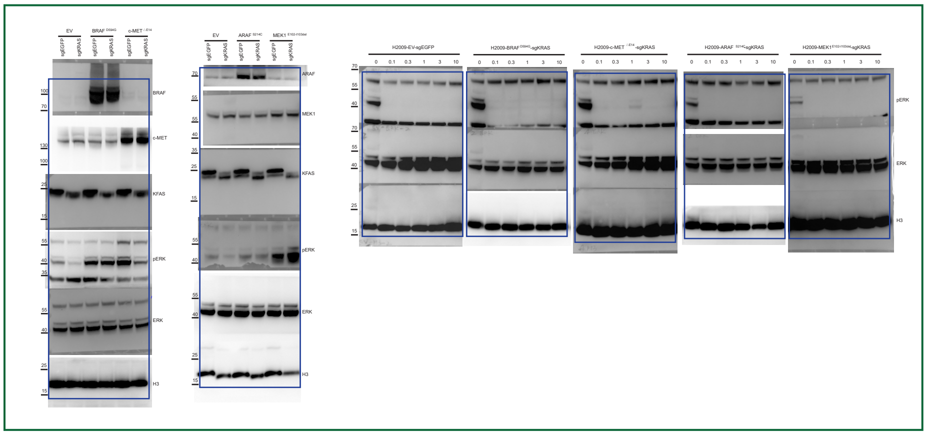

Supplementary Figure 14c

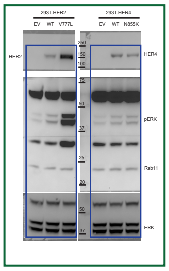

Supplementary Figure 16. All uncropped blots data.

Supplementary Table 1: Sequencing statistics

Sample ID	Description	Tissue	Coverage	>20X Coverage	Somatic SNVs	Mutation Rate	Protein-altering SNVs	Somatic Indels	Somatic CNVs	CNV gain	CNV loss
RJLC1-N	Normal	Frozen	236.22	99.30%	NA	NA	NA	NA	NA	NA	NA
RJLC1-T1R1	Tumor	Frozen	262.55	99.30%	180	0.658	28	1	48	36	12
RJLC1-T1R2	Tumor	Frozen	295.77	99.40%	232	0.897	35	5	15	14	1
RJLC1-T1R3	Tumor	Frozen	281.14	99.40%	219	0.877	33	3	25	23	2
RJLC1-T1R4	Tumor	Frozen	281.36	99.40%	229	0.797	32	3	25	25	0
RJLC1-T2	Tumor	Frozen	249.27	99.30%	296	0.758	26	2	199	199	0
RJLC2-N	Normal	Frozen	249.33	99.40%	NA	NA	NA	NA	NA	NA	NA
RJLC2-T1	Tumor	Frozen	256.62	99.40%	173	0.578	21	2	26	26	0
RJLC2-T2	Tumor	Frozen	265.06	99.40%	177	0.458	20	3	34	32	2
RJLC2-T3	Tumor	Frozen	263.22	99.40%	167	0.538	20	2	49	47	2
RJLC3-N	Normal	Frozen	243.74	99.40%	NA	NA	NA	NA	NA	NA	NA
RJLC3-T1	Tumor	Frozen	234.68	99.50%	443	0.857	35	4	88	88	0
RJLC3-T2	Tumor	Frozen	211.45	99.10%	525	0.818	30	9	58	44	14
RJLC3-T3	Tumor	Frozen	225.54	99.30%	224	0.797	31	13	73	72	1
RJLC4-N	Normal	Frozen	243.43	99.40%	NA	NA	NA	NA	NA	NA	NA
RJLC4-T1R1	Tumor	Frozen	221.46	99.30%	679	1.500	59	11	311	308	3
RJLC4-T1R2	Tumor	Frozen	235.33	99.40%	531	1.375	51	10	747	714	33
RJLC4-T1R3	Tumor	Frozen	214.65	99.30%	441	1.356	52	8	282	279	3
RJLC4-T2	Tumor	Frozen	204.98	99.20%	495	0.619	28	1	32	30	2
RJLC4-T3	Tumor	Frozen	212.78	99.30%	662	0.638	26	3	130	102	28

Supplementary Table 2: Driver mutation analysis

Hugo_Symbol	Chromosome	Position	Ref_allele	Alt_allele	Variant_Classification	AAChange	dbSNP_RS	COSMIC	CGC	BertVogelstein125	Comprehensive435	SMG127	Tumor_Sample_Name	ljb23_sift	ljb23_pp2hvar	ljb23_pp2hdiv	ljb23_mt
DST	6	56505297	C	T	Missense_Mutation	DST:NM_001723:exon4:c.G523A:p.G175R	.	.	-	-	Candidate driver	-	RJLC1-T1R1	0.58,0.42,T	1.0,D	1.0,D	0.833,0.833,D
EGFR	7	55259515	T	G	Missense_Mutation	EGFR:NM_005228:exon21:c.T2573G:p.L858R	rs121434568	COSM6224	a, NSCLCN	Oncogene	High Confidence Drive	4.60%	RJLC1-T1R1	0,1.00,D	0.999,D	1.0,D	1.000,1.000,D
SPEG	2	220309843	G	A	Missense_Mutation	SPEG:NM_005876:exon3:c.G775A:p.A259T	.	OSM140563	-	-	Candidate driver	-	RJLC1-T1R1	0.21,0.79,T	0.011,B	0.166,B	1,1.0,D
RECQL4	8	145738379	C	T	Missense_Mutation	RECQL4:NM_004260:exon16:c.G2606A:p.G869D	.	.	n basal cell,	-	-	-	RJLC1-T1R1	.	.	.	.
STAG2	X	123220564	A	T	Missense_Mutation	STAG2:NM_006603:exon29:c.A3221T:p.K1074I	.	.	elanoma, E	TSG	High Confidence Drive	2.20%	RJLC1-T1R1	0.01,0.99,D	0.921,D	0.987,D	1.000,1.000,D
TOP1	20	39742614	C	T	Missense_Mutation	TOP1:NM_003286:exon15:c.C1457T:p.A486V	.	.	AML*	-	-	-	RJLC1-T1R1	0,1.00,D	0.998,D	1.0,D	1,1.0,D
PLCG1	20	39801264	G	A	Missense_Mutation	PLCG1:NM_002660:exon26:c.G3109A:p.A1037T	.	.	ngiosarcom	-	High Confidence Drive	-	RJLC1-T1R1	0,1.00,D	0.998,D	1.0,D	1,1.0,D
MYH11	16	15841968	C	T	Missense_Mutation	MYH11:NM_002474:exon17:c.G2116A:p.E706K	.	.	AML	-	-	-	RJLC1-T1R2	0,1.00,D	0.984,D	1.0,D	1,1.0,D
DST	6	56505297	C	T	Missense_Mutation	DST:NM_001723:exon4:c.G523A:p.G175R	.	.	-	-	Candidate driver	-	RJLC1-T1R2	0.58,0.42,T	1.0,D	1.0,D	0.833,0.833,D
PLCG1	20	39801082	G	C	Missense_Mutation	PLCG1:NM_002660:exon26:c.G2927C:p.C976S	.	.	ngiosarcom	-	High Confidence Drive	-	RJLC1-T1R2	0.75,0.25,T	0.03,B	0.073,B	1,1.0,D
EGFR	7	55259515	T	G	Missense_Mutation	EGFR:NM_005228:exon21:c.T2573G:p.L858R	rs121434568	COSM6224	a, NSCLCN	Oncogene	High Confidence Drive	4.60%	RJLC1-T1R2	0,1.00,D	0.999,D	1.0,D	1.000,1.000,D
SPEG	2	220309843	G	A	Missense_Mutation	SPEG:NM_005876:exon3:c.G775A:p.A259T	.	OSM140563	-	-	Candidate driver	-	RJLC1-T1R2	0.21,0.79,T	0.011,B	0.166,B	1,1.0,D
RECQL4	8	145738379	C	T	Missense_Mutation	RECQL4:NM_004260:exon16:c.G2606A:p.G869D	.	.	n basal cell,	-	-	-	RJLC1-T1R2	.	.	.	.
STAG2	X	123220564	A	T	Missense_Mutation	STAG2:NM_006603:exon29:c.A3221T:p.K1074I	.	.	elanoma, E	TSG	High Confidence Drive	2.20%	RJLC1-T1R2	0.01,0.99,D	0.921,D	0.987,D	1.000,1.000,D
TOP1	20	39742614	C	T	Missense_Mutation	TOP1:NM_003286:exon15:c.C1457T:p.A486V	.	.	AML*	-	-	-	RJLC1-T1R2	0,1.00,D	0.998,D	1.0,D	1,1.0,D
DST	6	56505297	C	T	Missense_Mutation	DST:NM_001723:exon4:c.G523A:p.G175R	.	.	-	-	Candidate driver	-	RJLC1-T1R3	0.58,0.42,T	1.0,D	1.0,D	0.833,0.833,D
PTPRC	1	198717250	T	G	Missense_Mutation	PTPRC:NM_080921:exon24:c.T2377G:p.Y793D	.	.	T-ALL	-	-	-	RJLC1-T1R3	0.01,0.99,D	0.993,D	1.0,D	1.000,1.000,D
EGFR	7	55259515	T	G	Missense_Mutation	EGFR:NM_005228:exon21:c.T2573G:p.L858R	rs121434568	COSM6224	a, NSCLCN	Oncogene	High Confidence Drive	4.60%	RJLC1-T1R3	0,1.00,D	0.999,D	1.0,D	1.000,1.000,D
SPEG	2	220309843	G	A	Missense_Mutation	SPEG:NM_005876:exon3:c.G775A:p.A259T	.	OSM140563	-	-	Candidate driver	-	RJLC1-T1R3	0.21,0.79,T	0.011,B	0.166,B	1,1.0,D
RECQL4	8	145738379	C	T	Missense_Mutation	RECQL4:NM_004260:exon16:c.G2606A:p.G869D	.	.	n basal cell,	-	-	-	RJLC1-T1R3	.	.	.	.
STAG2	X	123220564	A	T	Missense_Mutation	STAG2:NM_006603:exon29:c.A3221T:p.K1074I	.	.	elanoma, E	TSG	High Confidence Drive	2.20%	RJLC1-T1R3	0.01,0.99,D	0.921,D	0.987,D	1.000,1.000,D
TOP1	20	39742614	C	T	Missense_Mutation	TOP1:NM_003286:exon15:c.C1457T:p.A486V	.	.	AML*	-	-	-	RJLC1-T1R3	0,1.00,D	0.998,D	1.0,D	1,1.0,D
MYH11	16	15841968	C	T	Missense_Mutation	MYH11:NM_002474:exon17:c.G2116A:p.E706K	.	.	AML	-	-	-	RJLC1-T1R4	0,1.00,D	0.984,D	1.0,D	1,1.0,D
DST	6	56505297	C	T	Missense_Mutation	DST:NM_001723:exon4:c.G523A:p.G175R	.	.	-	-	Candidate driver	-	RJLC1-T1R4	0.58,0.42,T	1.0,D	1.0,D	0.833,0.833,D
EGFR	7	55259515	T	G	Missense_Mutation	EGFR:NM_005228:exon21:c.T2573G:p.L858R	rs121434568	COSM6224	a, NSCLCN	Oncogene	High Confidence Drive	4.60%	RJLC1-T1R4	0,1.00,D	0.999,D	1.0,D	1.000,1.000,D
SPEG	2	220309843	G	A	Missense_Mutation	SPEG:NM_005876:exon3:c.G775A:p.A259T	.	OSM140563	-	-	Candidate driver	-	RJLC1-T1R4	0.21,0.79,T	0.011,B	0.166,B	1,1.0,D
RECQL4	8	145738379	C	T	Missense_Mutation	RECQL4:NM_004260:exon16:c.G2606A:p.G869D	.	.	n basal cell,	-	-	-	RJLC1-T1R4	.	.	.	.
STAG2	X	123220564	A	T	Missense_Mutation	STAG2:NM_006603:exon29:c.A3221T:p.K1074I	.	.	elanoma, E	TSG	High Confidence Drive	2.20%	RJLC1-T1R4	0.01,0.99,D	0.921,D	0.987,D	1.000,1.000,D
TOP1	20	39742614	C	T	Missense_Mutation	TOP1:NM_003286:exon15:c.C1457T:p.A486V	.	.	AML*	-	-	-	RJLC1-T1R4	0,1.00,D	0.998,D	1.0,D	1,1.0,D
EGFR	7	55259515	T	G	Missense_Mutation	EGFR:NM_005228:exon21:c.T2573G:p.L858R	rs121434568	COSM6224	a, NSCLCN	Oncogene	High Confidence Drive	4.60%	RJLC1-T2	0,1.00,D	0.999,D	1.0,D	1.000,1.000,D
BCL9L	11	118773748	G	A	Missense_Mutation	BCL9L:NM_182557:exon5:c.C785T:p.S262F	.	.	-	-	Candidate driver	-	RJLC2-T1	0,1.00,D	0.191,B	0.662,P	1.000,1.000,D
BRAF	7	140453154	T	C	Missense_Mutation	BRAF:NM_004333:exon15:c.A1781G:p.D594G	rs12191333	COSM467	ilocytic astr	Oncogene	High Confidence Drive	1.50%	RJLC2-T1	0,1.00,D	0.983,D	0.994,D	1,1.0,D
USP9X	X	41058006	A	C	Splice_Site	NM_001039590:exon30:c.4603+3A>C	.	.	-	-	Candidate driver	2.10%	RJLC2-T1	.	.	.	.
KRAS	12	25398284	C	T	Missense_Mutation	KRAS:NM_004985:exon2:c.G35A:p.G12D	rs12191352	1135366,CO	ng, thyroid,	Oncogene	High Confidence Drive	6.70%	RJLC2-T2	0,1.00,D	0.361,B	0.517,P	1,1.0,D
RAB11FIP5	2	73315825	G	C	Missense_Mutation	RAB11FIP5:NM_015470:exon3:c.C921G:p.S307R	.	.	-	-	Candidate driver	-	RJLC2-T3	0,1.00,D	0.986,D	1.0,D	0.994,0.994,D
MET	7	116412043	G	C	Missense_Mutation	MET:NM_000245:exon14:c.G3028C:p.D1010H	.	.	neck squam	Oncogene	High Confidence Drive	-	RJLC2-T3	0.01,0.99,D	0.994,D	1.0,D	1,1.0,D
PDGFRB	5	149499063	C	T	Missense_Mutation	PDGFRB:NM_002609:exon20:c.G2765A:p.R922H	.	COSM21596	AML, CMML	-	-	-	RJLC2-T3	0,1.00,D	1.0,D	1.0,D	1.000,1.000,D
MAP2K1	15	66729093	CTGGAGA	C	In_Frame_Del	MAP2K1:NM_002755:exon3:c.302_307del:p.101_103del	.	COSM40499	melanoma,	Oncogene	High Confidence Drive	-	RJLC3-T1	.	.	.	.
CREB3L2	7	137686411	C	A	Missense_Mutation	CREB3L2:NM_001253775:exon1:c.G41T:p.W14L	.	.	myxoid sarc	-	-	-	RJLC3-T1	0.02,0.98,D	0.998,D	1.0,D	1,1.0,D
AKR1B1	7	134136481	C	A	Missense_Mutation	AKR1B1:NM_001628:exon2:c.G91T:p.A31S	.	.	-	Oncogene	High Confidence Drive	1.20%	RJLC3-T2	0.07,0.93,T	0.966,D	0.98,D	1.000,1.000,D
PIK3CA	3	178921572	G	A	Missense_Mutation	PIK3CA:NM_006218:exon5:c.G1054A:p.D352N	.	.	stric, gliobla	Oncogene	High Confidence Drive	17.80%	RJLC3-T2	0.5,0.50,T	0.284,B	0.606,P	1,1.0,D
SDHAF2	11	61205278	A	C	Missense_Mutation	SDHAF2:NM_017841:exon2:c.A218C:p.E73A	.	.	aragangliom	-	-	-	RJLC3-T2	0.01,0.99,D	0.936,D	0.996,D	1,1.0,D
ARAF	X	47426121	C	G	Missense_Mutation	ARAF:NM_001256196:exon7:c.C650G:p.S217C	.	.	-	-	High Confidence Drive	-	RJLC3-T2	0,1.00,D	0.926,D	0.997,D	1,1.0,D
PIK3CA	3	178922324	G	A	Missense_Mutation	PIK3CA:NM_006218:exon6:c.G1093A:p.E365K	.	6044,COSM	stric, gliobla	Oncogene	High Confidence Drive	17.80%	RJLC3-T2	0.62,0.38,T	0.285,B	0.536,P	1,1.0,D
PIK3CA	3	178928079	G	C	Missense_Mutation	PIK3CA:NM_006218:exon8:c.G1357C:p.E453Q	.	758,COSM1	stric, gliobla	Oncogene	High Confidence Drive	17.80%	RJLC3-T2	0.31,0.69,T	0.265,B	0.364,B	1,1.0,D
NAA15	4	140262101	A	C	Missense_Mutation	NAA15:NM_057175:exon4:c.A280C:p.K94Q	.	.	-	-	Candidate driver	-	RJLC3-T3	0.06,0.94,T	0.994,D	0.997,D	1,1.0,D
PLEC	8	145003289	G	T	Missense_Mutation	PLEC:NM_201378:exon25:c.C3200A:p.S1067Y	.	.	-	-	High Confidence Drive	-	RJLC3-T3	0,1.00,D	0.999,D	1.0,D	1,1.0,D
TAL2	9	108424781	A	G	Missense_Mutation	TAL2:NM_005421:exon1:c.A4G:p.T2A	.	.	T-ALL	-	-	-	RJLC3-T3	0.52,0.48,T	0.016,B	0.008,B	0.951,0.951,D
KRAS	12	25398285	C	A	Missense_Mutation	KRAS:NM_004985:exon2:c.G34T:p.G12C	rs12191353	1140136,CO	ng, thyroid,	Oncogene	High Confidence Drive	6.70%	RJLC3-T3	0.01,0.99,D	0.993,D	1.0,D	1,1.0,D
BRAF	7	140481400	TTCCAAATGA	T	In_Frame_Del	BRAF:NM_004333:exon11:c.1399_1407del:p.467_469del	.	.	ilocytic astr	Oncogene	High Confidence Drive	1.50%	RJLC3-T3	.	.	.	.
EGFR	7	55242464	AGGAATTAAGAGAAGC	A	In_Frame_Del	EGFR:NM_005228:exon19:c.2235_2249del:p.745_750del	rs121913421	COSM6223	a, NSCLCN	Oncogene	High Confidence Drive	4.60%	RJLC4-T1R1	.	.	.	.
VEZT	12	95693937	G	T	Splice_Site	NM_017599:exon12:c.1832-4G>T	.	.	-	-	Candidate driver	-	RJLC4-T1R1	.	.	.	.
KMT2C	7	151882672	C	A	Missense_Mutation	KMT2C:NM_170606:exon34:c.G5053T:p.A1685S	rs14584831	49561,COS	edulloblastor	TSG	High Confidence Drive	6.60%	RJLC4-T1R1	0.07,0.93,T	0.997,D	1.0,D	1,1.0,D
TET2	4	106157834	C	T	Missense_Mutation	TET2:NM_001127208:exon3:c.C2735T:p.A912V	.	.	MDS	TSG	High Confidence Drive	1.60%	RJLC4-T1R1	0.04,0.96,D	0.009,B	0.03,B	1,0.0,N
TP53	17	7576897	G	A	Nonsense_Mutation	TP53:NM_001126115:exon5:c.C553T:p.Q185X	.	OSM165085	her tumour t	TSG	High Confidence Drive	42.00%	RJLC4-T1R1	0.92,0.08,T	.	.	1,1,A
PTEN	10	89720649	A	T	Splice_Site	NM_000314:exon8:c.802-2A>T	.	COSM1373	harmartoma	TSG	High Confidence Drive	9.70%	RJLC4-T1R1	.	.	.	1,1.0,D
DST	6	56392520	A	C	Nonsense_Mutation	DST:NM_015548:exon49:c.T9824G:p.L3275X	.	.	-	-	Candidate driver	-	RJLC4-T1R1	0.5,0.50,T	.	.	1.000,1.000,A
MAP3K3	17	61744340	G	A	Missense_Mutation	MAP3K3:NM_002401:exon6:c.G422A:p.R141Q	.	.	-	-	High Confidence Drive	-	RJLC4-T1R1	0.27,0.73,T	0.145,B	0.757,P	1.000,1.000,D
CUX1	7	101747612	G	T	Splice_Site	NM_001202543:exon6:c.440-4G>T	.	.	lorectal, AM	-	Candidate driver	-	RJLC4-T1R1	.	.	.	.
TP53	17	7577535	C	T	Missense_Mutation	TP53:NM_001126115:exon3:c.G350A:p.R117K	.	OSM129832	her tumour t	TSG	High Confidence Drive	42.00%	RJLC4-T1R1	0,1.00,D	0.961,D	0.995,D	0.999,0.999,D
EGFR	7	55242464	AGGAATTAAGAGAAGC	A	In_Frame_Del	EGFR:NM_005228:exon19:c.2235_2249del:p.745_750del	rs121913421	COSM6223	a, NSCLCN	Oncogene	High Confidence Drive	4.60%	RJLC4-T1R2	.	.	.	.
TP53	17	7576897	G	A	Nonsense_Mutation	TP53:NM_001126115:exon5:c.C553T:p.Q185X	.	OSM165085	her tumour t	TSG	High Confidence Drive	42.00%	RJLC4-T1R2	0.92,0.08,T	.	.	1,1,A
MAP3K3	17	61744340	G	A	Missense_Mutation	MAP3K3:NM_002401:exon6:c.G422A:p.R141Q	.	.	-	-	High Confidence Drive	-	RJLC4-T1R2	0.27,0.73,T	0.145,B	0.757,P	1.000,1.000,D
TET2	4	106157834	C	T	Missense_Mutation	TET2:NM_001127208:exon3:c.C2735T:p.A912V	.	.	MDS	TSG	High Confidence Drive	1.60%	RJLC4-T1R2	0.04,0.96,D	0.009,B	0.03,B	1,0.0,N
TP53	17	7577535	C	T	Missense_Mutation	TP53:NM_001126115:exon3:c.G350A:p.R117K	.	OSM129832	her tumour t	TSG	High Confidence Drive	42.00%	RJLC4-T1R2	0,1.00,D	0.961,D	0.995,D	0.999,0.999,D
EGFR	7	55242464	AGGAATTAAGAGAAGC	A	In_Frame_Del	EGFR:NM_005228:exon19:c.2235_2249del:p.745_750del	rs121913421	COSM6223	a, NSCLCN	Oncogene	High Confidence Drive	4.60%	RJLC4-T1R3	.	.	.	.
ASPM	1	197060124	A	C	Nonsense_Mutation	ASPM:NM_001206846:exon22:c.T4737G:p.Y1579X	rs143931757	.	-	-	High Confidence Drive	-	RJLC4-T1R3	1,0.00,T	.	.	1,1,A
TP53	17	7576897	G	A	Nonsense_Mutation	TP53:NM_001126115:exon5:c.C553T:p.Q185X	.	OSM165085	her tumour t	TSG	High Confidence Drive	42.00%	RJLC4-T1R3	0.92,0.08,T	.	.	1,1,A
MAP3K3	17	61744340	G	A	Missense_Mutation	MAP3K3:NM_002401:exon6:c.G422A:p.R141Q	.	.	-	-	High Confidence Drive	-	RJLC4-T1R3	0.27,0.73,T	0.145,B	0.757,P	1.000,1.000,D
TET2	4	106157834	C	T	Missense_Mutation	TET2:NM_001127208:exon3:c.C2735T:p.A912V	.	.	MDS	TSG	High Confidence Drive	1.60%	RJLC4-T1R3	0.04,0.96,D	0.009,B	0.03,B	1,0.0,N
TP53	17	7577535	C	T	Missense_Mutation	TP53:NM_001126115:exon3:c.G350A:p.R117K	.	OSM129832	her tumour t	TSG	High Confidence Drive	42.00%	RJLC4-T1R3	0,1.00,D	0.961,D	0.995,D	0.999,0.999,D
ASPM	1	197060124	A	C	Nonsense_Mutation	ASPM:NM_001206846:exon22:c.T4737G:p.Y1579X	rs143931757	.	-	-	High Confidence Drive	-	RJLC4-T2	1,0.00,T	.	.	1,1,A
CNOT1	16	58585156	A	C	Missense_Mutation	CNOT1:NM_001265612:exon24:c.T3207G:p.N1069K	.	.	-	-	Candidate driver	-	RJLC4-T2	0.12,0.88,T	0.989,D	1.0,D	1,1.0,D
ERBB2	17	37880981	A	AGCATACGTGATG	In_Frame_Ins	ERBB2:NM_001289937:exon20:c.2310_2311insGCATACGTGATG:p.E770delinsEAYVM	.	.	er tumour ty	Oncogene	High Confidence Drive	-	RJLC4-T2	.	.	.	.
BRAF	7	140434574	C	A	Splice_Site	NM_004333:exon19:c.2128-4G>T	.	.	ilocytic astr	Oncogene	High Confidence Drive	1.50%	RJLC4-T3	.	.	.	.
ERBB2	17	37880981	A	AGCATACGTGATG	In_Frame_Ins	ERBB2:NM_001289937:exon20:c.2310_2311insGCATACGTGATG:p.E770delinsEAYVM	.	.	er tumour ty	Oncogene	High Confidence Drive	-	RJLC4-T3	.	.	.	.

Supplementary Table 3: Driver mutation prevalence in lung cancer samples

Driver genes	Prevalence in pan-lung cancer of TCGA (n=1144 samples)	Prevalence in LUAC of TCGA (n=230 samples)	Mutations identified in this study	Mutated tumors	Mutations functionally implicated in lung tumorigenesis
KRAS	23.0%	36.0%	G12C; G12D	RJLC2-T2; RJLC3-T3	Yes
EGFR	14.0%	17.0%	L858R; E746-A750 deletion	RJLC1-T1; RJLC1-T2; RJLC4-T1	Yes
ERBB2	4.0%	5.0%	Y772-A775 duplication	RJLC4-T2; RJLC4-T3	Yes
MET	5.0%	12.0%	Exon 14 splice site	RJLC2-T3	Yes
ARAF	2.7%	2.6%	S214C	RJLC3-T2	Yes
BRAF	7.0%	11.0%	D594G	RJLC2-T1	No
MAP2K1	1.7%	1.7%	E102-I103 deletion	RJLC3-T1	No

Supplementary Table 4: Neoantigen repertoires predicted by NetMHCpan algorithm

Sample	MutatatedGene	Mutation information	Chr	Position	ref	alt	T_vaf	T(DV/DN)	N(DV/DN)	HLA	wildPeptide	Neoantigens	wildPeptide-Aff(nM)	Neoantigens-Aff(nM)
RJLC1-T1	NBPF10	NM_001039703:exon6:c.T836G:p.L279W	1	145299787	T	G	0.12	3:22	0:17	HLA-A*11:01	SAGPLSSEK	SAGPWSSEK	135.7	73.8
RJLC1-T1	SPEG	NM_005876:exon3:c.G775A:p.A259T	2	220309843	G	A	0.171	0.855555556	0.084027778	HLA-A*11:01	SAFSLYRGR	STFSLYRGR	309.5	42.5
RJLC1-T1	PRR21	NM_001080835:exon1:c.C847T:p.P283S	2	240981553	G	A	0.167	3:15	1:21	HLA-A*11:01	GPSSMPLHPR	GSSSMPLHPR	134.5	76.5
RJLC1-T1	PRR21	NM_001080835:exon1:c.C847T:p.P283S	2	240981553	G	A	0.167	3:15	1:21	HLA-A*11:01	PSSMPLHPR	SSSMPLHPR	1123.5	27
RJLC1-T1	PGK2	NM_138733:exon1:c.A679T:p.M227L	6	49754222	T	A	0.08	26:300	0.157638889	HLA-A*11:01	QLIKNMLDK	QLIKNLLDK	245.4	233.4
RJLC1-T1	RSPH3	NM_031924:exon6:c.C1273A:p.P425T	6	159401818	G	T	0.118	0.984027778	0.088888889	HLA-A*11:01	SGYFYDPIER	SGYFYDTIER	556.6	313.5
RJLC1-T1	RECQL4	NM_004260:exon16:c.G2606A:p.G869D	8	145738379	C	T	0.175	37:174	0.122222222	HLA-A*11:01	AVGGERPVPK	AVDGERPVPK	135.2	99.8
RJLC1-T1	SERPINA5	NM_000624:exon3:c.C452T:p.T151M	14	95054151	C	T	0.151	50:281	0.289583333	HLA-A*11:01	TFVSAMKTLY	TFVSAMKMLY	1622.6	249.1
RJLC1-T1	SERPINA5	NM_000624:exon3:c.C452T:p.T151M	14	95054151	C	T	0.151	50:281	0.289583333	HLA-A*11:01	FVSAMKTLY	FVSAMKMLY	1495	275.2
RJLC1-T1	PLA2G4D	NM_178034:exon3:c.G160A:p.A54T	15	42379593	C	T	0.036	0.6	0.154861111	HLA-A*11:01	LQLSTAPGMK	LQLSTTPGMK	402.1	354.6
RJLC1-T1	PLA2G4D	NM_178034:exon3:c.G160A:p.A54T	15	42379593	C	T	0.036	0.6	0.154861111	HLA-A*11:01	TAPGMKFKTK	TTPGMKFKTK	1452.9	244
RJLC1-T1	TOP1	NM_003286:exon15:c.C1457T:p.A486V	20	39742614	C	T	0.118	0.65625	0.051388889	HLA-A*11:01	KLALRAGNEK	KLVLRAGNEK	326.3	251.8
RJLC1-T1	TOP1	NM_003286:exon15:c.C1457T:p.A486V	20	39742614	C	T	0.118	0.65625	0.051388889	HLA-A*11:01	LALRAGNEK	LVLRAGNEK	453	101.2
RJLC1-T1	CHM	NM_000390:exon13:c.G1570C:p.V524L	X	85134009	C	G	0.064	0.311111111	0.045138889	HLA-A*11:01	SVVQKLFVPY	SVLQKLFVPY	97.4	88.7
RJLC1-T1	STAG2	NM_006603:exon29:c.A3221T:p.K1074I	X	123220564	A	T	0.156	35:189	0.113194444	HLA-A*11:01	GSTVRSKKSK	GSTVRSKISK	2400.8	112
RJLC1-T1	STAG2	NM_006603:exon29:c.A3221T:p.K1074I	X	123220564	A	T	0.156	35:189	0.113194444	HLA-A*11:01	SKKSKPSTGK	SKISKPSTGK	18407.8	118.9
RJLC1-T1	STAG2	NM_006603:exon29:c.A3221T:p.K1074I	X	123220564	A	T	0.156	35:189	0.113194444	HLA-A*11:01	STVRSKKSK	STVRSKISK	239.6	18.3
RJLC1-T1	STAG2	NM_006603:exon29:c.A3221T:p.K1074I	X	123220564	A	T	0.156	35:189	0.113194444	HLA-A*11:01	KKSKPSTGK	KISKPSTGK	11479.2	44.9
RJLC1-T1	CYBRD1	NM_001256909:exon4:c.C413T:p.P138L	2	172411063	C	T	0.032	0.438888889	0.129166667	HLA-A*24:02	AYSTFPPEGVF	AYSTFPLEGVF	891	380.5
RJLC1-T1	SERPINA5	NM_000624:exon3:c.C452T:p.T151M	14	95054151	C	T	0.151	50:281	0.289583333	HLA-A*24:02	KTLYLADTF	KMLYLADTF	1044.4	147.1
RJLC1-T1	RALGAPA2	NM_020343:exon10:c.G1222T:p.V408L	20	20610018	C	A	0.043	0.343055556	0.063194444	HLA-A*24:02	RGYVNFVNEV	RGYVNFVNEL	383.8	180.3
RJLC1-T1	RALGAPA2	NM_020343:exon10:c.G1222T:p.V408L	20	20610018	C	A	0.043	0.343055556	0.063194444	HLA-A*24:02	GYVNFVNEVF	GYVNFVNELF	78.7	36.7
RJLC1-T1	RALGAPA2	NM_020343:exon10:c.G1222T:p.V408L	20	20610018	C	A	0.043	0.343055556	0.063194444	HLA-A*24:02	GYVNFVNEV	GYVNFVNEL	667.6	342.8
RJLC1-T1	CYBRD1	NM_001256909:exon4:c.C413T:p.P138L	2	172411063	C	T	0.032	0.438888889	0.129166667	HLA-B*40:01	PEGVFVNTL	LEGVFVNTL	1314.5	32.8
RJLC1-T1	WNT9B	NM_003396:exon3:c.C446T:p.P149L	17	44952578	C	T	0.038	0.652777778	0.185416667	HLA-B*40:01	MERCTCDDSP	MERCTCDDSL	19257.5	155.1
RJLC1-T1	CHM	NM_000390:exon13:c.G1570C:p.V524L	X	85134009	C	G	0.064	0.311111111	0.045138889	HLA-B*40:01	REDLESVV	REDLESVL	1546.7	284.2
RJLC1-T1	EPM2AIP1	NM_014805:exon1:c.C1024T:p.L342F	3	37033545	G	A	0.184	99:438	0.311805556	HLA-C*07:02	LRRGKTLKL	FRRGKTLKL	158	31
RJLC1-T2	NBPF10	NM_001039703:exon6:c.T836G:p.L279W	1	145299787	T	G	0.19	0.178472222	0.011805556	HLA-A*11:01	SAGPLSSEK	SAGPWSSEK	135.7	73.8
RJLC1-T2	TAF1B	NM_005680:exon13:c.C1317A:p.D439E	2	10059215	C	A	0.036	0.6	0.203472222	HLA-A*11:01	KPLYYSFVDK	KPLYYSFVEK	232.4	76.7
RJLC1-T2	TAF1B	NM_005680:exon13:c.C1317A:p.D439E	2	10059215	C	A	0.036	0.6	0.203472222	HLA-A*11:01	PLYYSFVDK	PLYYSFVEK	764.6	221.5
RJLC1-T2	MANEA	NM_024641:exon5:c.G1283T:p.R428L	6	96054175	G	T	0.034	0.733333333	0.214583333	HLA-A*11:01	NTVYLDYRPHK	NTVYLDYLPHK	1243.7	300.6
RJLC1-T2	MANEA	NM_024641:exon5:c.G1283T:p.R428L	6	96054175	G	T	0.034	0.733333333	0.214583333	HLA-A*11:01	TVYLDYRPHK	TVYLDYLPHK	63.8	19.4
RJLC1-T2	ERCC6L2	NM_001010895:exon6:c.G1099T:p.A367S	9	98678624	G	T	0.079	0.397916667	0.063888889	HLA-A*11:01	RKAMQRLAKK	RKSMQRLAKK	121.6	53.1
RJLC1-T2	ERCC6L2	NM_001010895:exon6:c.G1099T:p.A367S	9	98678624	G	T	0.079	0.397916667	0.063888889	HLA-A*11:01	ATGRKAMQR	ATGRKSMQR	266.2	217.4
RJLC1-T2	ERCC6L2	NM_001010895:exon6:c.G1099T:p.A367S	9	98678624	G	T	0.079	0.397916667	0.063888889	HLA-A*11:01	KAMQRLAKK	KSMQRLAKK	29.5	14
RJLC1-T2	TFF1	NM_003225:exon2:c.C140T:p.T47M	21	43783462	G	A	0.091	0.486111111	0.077083333	HLA-A*11:01	GVTPSQCANK	GVMPSQCANK	187.9	30.4
RJLC1-T2	F9	NM_000133:exon8:c.G1012C:p.D338H	X	138643856	G	C	0.03	0.966666667	0.333333333	HLA-A*11:01	YVTPICIADK	YVTPICIAHK	137	92
RJLC1-T2	F9	NM_000133:exon8:c.G1012C:p.D338H	X	138643856	G	C	0.03	0.966666667	0.333333333	HLA-A*11:01	VTPICIADK	VTPICIAHK	410.7	179.6
RJLC1-T2	DGAT2	NM_001253891:exon2:c.T143G:p.L48R	11	75501254	T	G	0.106	0.332638889	0.044444444	HLA-A*24:02	LMYIFCTDCW	RMYIFCTDCW	226.1	83.2
RJLC1-T2	SLC9A7	NM_001257291:exon15:c.G1790T:p.W597L	X	46480445	C	A	0.038	0.413888889	0.139583333	HLA-A*24:02	WIFRLWYSF	LIFRLWYSF	424.4	208.6
RJLC1-T2	F9	NM_000133:exon8:c.G1012C:p.D338H	X	138643856	G	C	0.03	0.966666667	0.333333333	HLA-A*24:02	DKEYTNIFLKF	HKEYTNIFLKF	550.5	258.5
RJLC1-T2	ADD3	NM_001121:exon2:c.A119G:p.N40S	10	111860530	A	G	0.046	0.504861111	0.11875	HLA-B*40:01	RERNMSPDL	RERSMSPDL	195.6	138.1
RJLC1-T2	SLC9A7	NM_001257291:exon15:c.G1790T:p.W597L	X	46480445	C	A	0.038	0.413888889	0.139583333	HLA-B*40:01	KQESAWIFRL	KQESALIFRL	502.6	314.5
RJLC1-T2	SLC9A7	NM_001257291:exon15:c.G1790T:p.W597L	X	46480445	C	A	0.038	0.413888889	0.139583333	HLA-B*40:01	QESAWIFRL	QESALIFRL	31.4	21.9
RJLC1-T2	KIF26B	NM_018012:exon10:c.G2107C:p.G703R	1	245809431	G	C	0.101	0.430555556	0.069444444	HLA-C*07:02	GGRSRLHLI	GRRSRLHLI	7981.9	415.2
RJLC1-T2	DLK1	NM_003836:exon5:c.G779A:p.S260N	14	101200860	G	A	0.032	0.377083333	0.138888889	HLA-C*07:02	TRLPSGYGL	TRLPNGYGL	733.2	420.6
RJLC2-T1	BRAF	NM_004333:exon15:c.A1781G:p.D594G	7	140453154	T	C	0.075	0.301388889	0.065277778	HLA-A*11:01	GDFGLATVK	GGFGLATVK	3782.6	396.7
RJLC2-T1	CSMD3	NM_052900:exon3:c.G481T:p.V161F	8	114290854	C	A	0.219	61:217	0.147222222	HLA-A*11:01	SDFAVSAHGFK	SDFAFSAHGFK	1022.9	272.7
RJLC2-T1	CSMD3	NM_052900:exon3:c.G481T:p.V161F	8	114290854	C	A	0.219	61:217	0.147222222	HLA-A*11:01	DFAVSAHGFK	DFAFSAHGFK	1152	290
RJLC2-T1	CSMD3	NM_052900:exon3:c.G481T:p.V161F	8	114290854	C	A	0.219	61:217	0.147222222	HLA-A*11:01	FAVSAHGFK	FAFSAHGFK	112.5	28.7
RJLC2-T1	F7	NM_001267554:exon6:c.C871T:p.R291W	13	113773044	C	T	0.127	29:199	0.193055556	HLA-A*11:01	MTQDCLQQSRK	MTQDCLQQSWK	660.1	340.9
RJLC2-T1	RYR1	NM_001042723:exon90:c.A12865G:p.T4289A	19	39055854	A	G	0.286	0.173611111	0.008333333	HLA-A*11:01	ATAAAGATAR	ATAAAGAAAR	184.8	184.6
RJLC2-T1	SLC5A1	NM_001256314:exon7:c.G310A:p.A104T	22	32480452	G	A	0.106	25:211	0.180555556	HLA-A*11:01	EVGGYDAFMEK	EVGGYDTFMEK	512.8	496.3
RJLC2-T1	SLC5A1	NM_001256314:exon7:c.G310A:p.A104T	22	32480452	G	A	0.106	25:211	0.180555556	HLA-A*11:01	GYDAFMEKYMK	GYDTFMEKYMK	1500	268.1
RJLC2-T1	SLC5A1	NM_001256314:exon7:c.G310A:p.A104T	22	32480452	G	A	0.106	25:211	0.180555556	HLA-A*11:01	VGGYDAFMEK	VGGYDTFMEK	221.7	202.2
RJLC2-T1	SLC5A1	NM_001256314:exon7:c.G310A:p.A104T	22	32480452	G	A	0.106	25:211	0.180555556	HLA-A*11:01	YDAFMEKYMK	YDTFMEKYMK	166.8	43.2
RJLC2-T1	SLC5A1	NM_001256314:exon7:c.G310A:p.A104T	22	32480452	G	A	0.106	25:211	0.180555556	HLA-A*11:01	DAFMEKYMK	DTFMEKYMK	2400.8	398.1
RJLC2-T1	IL13RA2	NM_000640:exon9:c.C1090T:p.R364C	X	114239786	G	A	0.12	0.654861111	0.08125	HLA-A*11:01	LLLRKPNTYPK	LLLCKPNTYPK	326.5	286.4
RJLC2-T1	IL13RA2	NM_000640:exon9:c.C1090T:p.R364C	X	114239786	G	A	0.12	0.654861111	0.08125	HLA-A*11:01	LLRKPNTYPK	LLCKPNTYPK	210.9	72.5
RJLC2-T1	CSMD3	NM_052900:exon3:c.G481T:p.V161F	8	114290854	C	A	0.219	61:217	0.147222222	HLA-A*24:02	SDFAVSAHGF	SDFAFSAHGF	763.8	347.6
RJLC2-T1	CSMD3	NM_052900:exon3:c.G481T:p.V161F	8	114290854	C	A	0.219	61:217	0.147222222	HLA-B*15:02	FAVSAHGFKVY	FAFSAHGFKVY	271	135.8
RJLC2-T1	CSMD3	NM_052900:exon3:c.G481T:p.V161F	8	114290854	C	A	0.219	61:217	0.147222222	HLA-B*15:02	SLRLTSDFAV	SLRLTSDFAF	11626.4	116.6
RJLC2-T1	CSMD3	NM_052900:exon3:c.G481T:p.V161F	8	114290854	C	A	0.219	61:217	0.147222222	HLA-B*15:02	VSAHGFKVYY	FSAHGFKVYY	776.1	190.9
RJLC2-T1	CSMD3	NM_052900:exon3:c.G481T:p.V161F	8	114290854	C	A	0.219	61:217	0.147222222	HLA-B*15:02	VSAHGFKVY	FSAHGFKVY	184.6	41
RJLC2-T1	IL13RA2	NM_000640:exon9:c.C1090T:p.R364C	X	114239786	G	A	0.12	0.654861111	0.08125	HLA-B*15:02	LLLRKPNTY	LLLCKPNTY	480.2	295.3
RJLC2-T1	BCL9L	NM_182557:exon5:c.C785T:p.S262F	11	118773748	G	A	0.133	0.923611111	0.140277778	HLA-C*14:02	GRADSILAY	GRADFILAY	287.4	155
RJLC2-T2	PCDHA1	NM_018900:exon1:c.G715T:p.A239S	5	140166590	G	T	0.085	30:322	0.249305556	HLA-A*11:01	APLFDQAVYR	SPLFDQAVYR	116.3	103
RJLC2-T2	RYR1	NM_001042723:exon90:c.A12865G:p.T4289A	19	39055854	A	G	0.357	5:09	0:12	HLA-A*11:01	ATAAAGATAR	ATAAAGAAAR	184.8	184.6
RJLC2-T2	CDK5RAP1	NM_001278167:exon5:c.C541G:p.L181V	20	31979951	G	C	0.048	0.333333333	0.102777778	HLA-A*11:01	ILGCMAERLK	IVGCMAERLK	674.9	291.6
RJLC2-T2	FAM65C	NM_080829:exon19:c.G2501A:p.R834K	20	49208945	C	T	0.091	0.971527778	0.122222222	HLA-A*11:01	ALLQLDGTPR	ALLQLDGTPK	2920.1	251.7
RJLC2-T2	CDK2	NM_001798:exon4:c.C469T:p.R157C	12	56362715	C	T	0.033	0.309027778	0.122916667	HLA-B*15:02	RAFGVPVRTY	RAFGVPVCTY	203.4	137.6
RJLC2-T2	MED13L	NM_015335:exon23:c.G5179T:p.V1727L	12	116418740	C	A	0.122	0.466666667	1:47	HLA-B*15:02	LQIVPCQYM	LQILPCQYM	325.2	312.4
RJLC2-T2	ZNF496	NM_032752:exon3:c.C167T:p.P56L	1	247492714	G	A	0.104	0.38125	0.050694444	HLA-C*14:02	FRYQEAAGP	FRYQEAAGL	17812.5	109.9
RJLC2-T3	AK5	NM_012093:exon6:c.A662T:p.N221I	1	77806102	A	T	0.039	0.880555556	0.258333333	HLA-A*11:01	ANQRLKERLLK	AIQRLKERLLK	11169.7	490.9
RJLC2-T3	AK5	NM_012093:exon6:c.A662T:p.N221I	1	77806102	A	T	0.039	0.880555556	0.258333333	HLA-A*11:01	VVFLACANQR	VVFLACAIQR	517.3	199.1
RJLC2-T3	AK5	NM_012093:exon6:c.A662T:p.N221I	1	77806102	A	T	0.039	0.880555556	0.258333333	HLA-A*11:01	FLACANQRLK	FLACAIQRLK	479.5	282.3
RJLC2-T3	AK5	NM_012093:exon6:c.A662T:p.N221I	1	77806102	A	T	0.039	0.880555556	0.258333333	HLA-A*11:01	LACANQRLK	LACAIQRLK	853.9	377.3
RJLC2-T3	RAB11FIP5	NM_015470:exon3:c.C921G:p.S307R	2	73315825	G	C	0.061	0.575694444	0.1375	HLA-A*11:01	KLFTHKRTYS	KLFTHKRTYR	15020.5	213.9
RJLC2-T3	HJURP	NM_001282963:exon5:c.G1193T:p.R398L	2	234749978	C	A	0.036	0.660416667	0.194444444	HLA-A*11:01	TRRLSLPSSK	TLRLSLPSSK	17822.4	141.8
RJLC2-T3	ERBB3	NM_001982:exon17:c.G1981A:p.G661S	12	56489516	G	A	0.043	0.799305556	0.213194444	HLA-A*11:01	MMLGGTFLYWR	MMLGSTFLYWR	592.4	358.5
RJLC2-T3	ERBB3	NM_001982:exon17:c.G1981A:p.G661S	12	56489516	G	A	0.043	0.799305556	0.213194444	HLA-A*11:01	FMMLGGTFLY	FMMLGSTFLY	243.1	160.7
RJLC2-T3	ERBB3	NM_001982:exon17:c.G1981A:p.G661S	12	56489516	G	A	0.043	0.799305556	0.213194444	HLA-A*11:01	MLGGTFLYWR	MLGSTFLYWR	371.5	165.7
RJLC2-T3	ERBB3	NM_001982:exon17:c.G1981A:p.G661S	12	56489516	G	A	0.043	0.799305556	0.213194444	HLA-A*11:01	GTFLYWRGRR	STFLYWRGRR	418.5	130.2
RJLC2-T3	ERBB3	NM_001982:exon17:c.G1981A:p.G661S	12	56489516	G	A	0.043	0.799305556	0.213194444	HLA-A*11:01	MMLGGTFLY	MMLGSTFLY	35.5	29.6
RJLC2-T3	ERBB3	NM_001982:exon17:c.G1981A:p.G661S	12	56489516	G	A	0.043	0.799305556	0.213194444	HLA-A*11:01	GTFLYWRGR	STFLYWRGR	135.5	46
RJLC2-T3	RYR1	NM_001042723:exon90:c.A12865G:p.T4289A	19	39055854	A	G	0.412	7:10	0:12	HLA-A*11:01	ATAAAGATAR	ATAAAGAAAR	184.8	184.6
RJLC2-T3	NRARP	NM_001004354:exon1:c.C166T:p.H56Y	9	140196215	G	A	0.046	0.953472222	0.236805556	HLA-A*24:02	LHQSVIDGNL	LYQSVIDGNL	16227.2	282.3
RJLC2-T3	PCNXL3	NM_032223:exon22:c.G3698T:p.S1233I	11	65395049	G	T	0.045	0.507638889	0.129166667	HLA-A*24:02	DYFLMSLLCS	DYFLMSLLCI	21915.4	344.9
RJLC2-T3	ERBB3	NM_001982:exon17:c.G1981A:p.G661S	12	56489516	G	A	0.043	0.799305556	0.213194444	HLA-A*24:02	VIFMMLGGTF	VIFMMLGSTF	245.5	72.6
RJLC2-T3	ERBB3	NM_001982:exon17:c.G1981A:p.G661S	12	56489516	G	A	0.043	0.799305556	0.213194444	HLA-A*24:02	IFMMLGGTFL	IFMMLGSTFL	1059	378.4
RJLC2-T3	ERBB3	NM_001982:exon17:c.G1981A:p.G661S	12	56489516	G	A	0.043	0.799305556	0.213194444	HLA-A*24:02	MMLGGTFLYW	MMLGSTFLYW	160.7	127.1
RJLC2-T3	ERBB3	NM_001982:exon17:c.G1981A:p.G661S	12	56489516	G	A	0.043	0.799305556	0.213194444	HLA-A*24:02	IFMMLGGTF	IFMMLGSTF	86.5	27.1
RJLC2-T3	ERBB3	NM_001982:exon17:c.G1981A:p.G661S	12	56489516	G	A	0.043	0.799305556	0.213194444	HLA-A*24:02	MLGGTFLYW	MLGSTFLYW	498	284.7
RJLC2-T3	DPY19L2	NM_173812:exon5:c.T610C:p.C204R	12	64041124	A	G	0.053	0.215972222	0:59	HLA-A*24:02	YCTFMGIMNLF	YRTFMGIMNLF	313.2	189.8
RJLC2-T3	DPY19L2	NM_173812:exon5:c.T610C:p.C204R	12	64041124	A	G	0.053	0.215972222	0:59	HLA-A*24:02	VIIASWYCTF	VIIASWYRTF	226.4	221.3
RJLC2-T3	DPY19L2	NM_173812:exon5:c.T610C:p.C204R	12	64041124	A	G	0.053	0.215972222	0:59	HLA-A*24:02	CTFMGIMNLF	RTFMGIMNLF	104.7	17.8
RJLC2-T3	DPY19L2	NM_173812:exon5:c.T610C:p.C204R	12	64041124	A	G	0.053	0.215972222	0:59	HLA-A*24:02	SWYCTFMGI	SWYRTFMGI	158.8	86.6
RJLC2-T3	TPI1	NM_001159287:exon1:c.C88T:p.L30F	12	6976707	C	T	0.049	0.330555556	0.083333333	HLA-B*15:02	DLQRLGSSAM	DFQRLGSSAM	377.6	279.1
RJLC2-T3	TPI1	NM_001159287:exon1:c.C88T:p.L30F	12	6976707	C	T	0.049	0.330555556	0.083333333	HLA-B*15:02	LQRLGSSAM	FQRLGSSAM	25.4	16.6
RJLC2-T3	ERBB3	NM_001982:exon17:c.G1981A:p.G661S	12	56489516	G	A	0.043	0.799305556	0.213194444	HLA-B*15:02	FMMLGGTFL	FMMLGSTFL	222.2	159.4
RJLC2-T3	ERBB3	NM_001982:exon17:c.G1981A:p.G661S	12	56489516	G	A	0.043	0.799305556	0.213194444	HLA-B*15:02	FMMLGGTF	FMMLGSTF	335	167.5
RJLC2-T3	HPS5	NM_007216:exon4:c.C43T:p.L15F	11	18332380	G	A	0.041	1.159027778	0.326388889	HLA-C*08:01	TALCWDTAI	TAFCWDTAI	420.4	171.6
RJLC2-T3	AK5	NM_012093:exon6:c.A662T:p.N221I	1	77806102	A	T	0.039	0.880555556	0.258333333	HLA-C*14:02	FLACANQRL	FLACAIQRL	207.3	166.7
RJLC2-T3	RAB11FIP5	NM_015470:exon3:c.C921G:p.S307R	2	73315825	G	C	0.061	0.575694444	0.1375	HLA-C*14:02	RTYSDEANQM	RTYRDEANQM	910.5	444.3
RJLC2-T3	RAB11FIP5	NM_015470:exon3:c.C921G:p.S307R	2	73315825	G	C	0.061	0.575694444	0.1375	HLA-C*14:02	TYSDEANQM	TYRDEANQM	48.9	19.6
RJLC2-T3	PDGFRB	NM_002609:exon20:c.G2765A:p.R922H	5	149499063	C	T	0.093	35:340	0.250694444	HLA-C*14:02	FYNAIKRGYRM	FYNAIKRGYHM	444.6	443.1
RJLC2-T3	PCNXL3	NM_032223:exon22:c.G3698T:p.S1233I	11	65395049	G	T	0.045	0.507638889	0.129166667	HLA-C*14:02	YFLMSLLCS	YFLMSLLCI	8048.5	496.8
RJLC2-T3	DPY19L2	NM_173812:exon5:c.T610C:p.C204R	12	64041124	A	G	0.053	0.215972222	0:59	HLA-C*14:02	SWYCTFMGIM	SWYRTFMGIM	395.6	207.7
RJLC2-T3	DPY19L2	NM_173812:exon5:c.T610C:p.C204R	12	64041124	A	G	0.053	0.215972222	0:59	HLA-C*14:02	YCTFMGIMNL	YRTFMGIMNL	3363.4	406.8
RJLC2-T3	DPY19L2	NM_173812:exon5:c.T610C:p.C204R	12	64041124	A	G	0.053	0.215972222	0:59	HLA-C*14:02	IASWYCTFM	IASWYRTFM	481.8	173.2
RJLC2-T3	DPY19L2	NM_173812:exon5:c.T610C:p.C204R	12	64041124	A	G	0.053	0.215972222	0:59	HLA-C*14:02	WYCTFMGIM	WYRTFMGIM	224.5	53.9

RJLC2-T3	DPY19L2	NM_173812:exon5:c.T610C:p.C204R	12	64041124	A	G	0.053	0.215972222	0:59	HLA-C*14:02	CTFMGIMNL	RTFMGIMNL	1170.1	350.4
RJLC3-T1	LRRC66	NM_001024611:exon1:c.A355G:p.N119D	4	52883425	T	C	0.156	25:135	0.159027778	HLA-A*02:01	NLSNNAIHSL	NLSNDAIHSL	1655.2	433.7
RJLC3-T1	VPS13B	NM_017890:exon44:c.G8148T:p.Q2716H	8	100821734	G	T	0.041	0.577083333	0.201388889	HLA-A*02:01	SLIIKVQQL	SLIIKVHQL	392.5	103.3
RJLC3-T1	MROH6	NM_001100878:exon3:c.C583G:p.R195G	8	144653856	G	C	0.062	0.3125	0.065277778	HLA-A*02:01	LLPRSLPADRV	LLPGSLPADRV	884.9	330.2
RJLC3-T1	MROH6	NM_001100878:exon3:c.C583G:p.R195G	8	144653856	G	C	0.062	0.3125	0.065277778	HLA-A*02:01	CALLPRSLPA	CALLPGSLPA	529.9	379.4
RJLC3-T1	MROH6	NM_001100878:exon3:c.C583G:p.R195G	8	144653856	G	C	0.062	0.3125	0.065277778	HLA-A*02:01	ALLPRSLPA	ALLPGSLPA	92.1	51.1
RJLC3-T1	GOLGA2	NM_004486:exon19:c.G1939A:p.V647M	9	131021523	C	T	0.226	14:48	0.047222222	HLA-A*02:01	LLQTQLVDQL	LLQTQLMDQL	539.3	493.7
RJLC3-T1	OR10G4	NM_001004462:exon1:c.G319A:p.G107R	11	123886600	G	A	0.048	0.777777778	0.292361111	HLA-A*02:01	GSTECFLYTV	RSTECFLYTV	571.3	431.3
RJLC3-T1	TRAPPC1	NM_021210:exon4:c.C395G:p.S132C	17	7833967	G	C	0.13	0.972916667	0.115277778	HLA-A*02:01	RLDSYVRSL	RLDCYVRSL	104.1	66.7
RJLC3-T1	PAGE2	NM_207339:exon4:c.C286G:p.P96A	X	55117857	C	G	0.029	0.456944444	0.243055556	HLA-A*02:01	GIMPTFDLTKV	GIMATFDLTKV	798.4	314.8
RJLC3-T1	PAGE2	NM_207339:exon4:c.C286G:p.P96A	X	55117857	C	G	0.029	0.456944444	0.243055556	HLA-A*02:01	IMPTFDLTKVL	IMATFDLTKVL	1878.2	448.4
RJLC3-T1	PAGE2	NM_207339:exon4:c.C286G:p.P96A	X	55117857	C	G	0.029	0.456944444	0.243055556	HLA-A*02:01	IMPTFDLTKV	IMATFDLTKV	88.8	18.9
RJLC3-T1	GLRA4	NM_001024452:exon7:c.C757T:p.R253W	X	102974161	G	A	0.154	26:143	0.136805556	HLA-A*02:01	HLERQMGYYL	HLEWQMGYYL	1389.5	51.8
RJLC3-T1	GLRA4	NM_001024452:exon7:c.C757T:p.R253W	X	102974161	G	A	0.154	26:143	0.136805556	HLA-A*02:01	RQMGYYLIQM	WQMGYYLIQM	208.3	144.8
RJLC3-T1	GLRA4	NM_001024452:exon7:c.C757T:p.R253W	X	102974161	G	A	0.154	26:143	0.136805556	HLA-A*02:01	RQMGYYLI	WQMGYYLI	1163.1	383.9
RJLC3-T1	ZRANB2	NM_005455:exon1:c.A35C:p.D12A	1	71546644	T	G	0.134	32:207	0.229166667	HLA-A*11:01	GDWICPDKK	GAWICPDKK	16072.8	219.8
RJLC3-T1	PITX1	NM_002653:exon2:c.A332G:p.N111S	5	134367036	T	C	0.202	65:256	0.200694444	HLA-A*11:01	RNRYPDMSMR	RSRYPDMSMR	6817.8	309.3
RJLC3-T1	TNIP1	NM_001252386:exon13:c.C1348G:p.L450V	5	150415157	G	C	0.134	26:168	0.111111111	HLA-A*11:01	TLSNAQLKAFK	TVSNAQLKAFK	108.2	91.2
RJLC3-T1	TNIP1	NM_001252386:exon13:c.C1348G:p.L450V	5	150415157	G	C	0.134	26:168	0.111111111	HLA-A*11:01	LSNAQLKAFK	VSNAQLKAFK	46.9	38.8
RJLC3-T1	WNK2	NM_006648:exon25:c.C6137A:p.T2046K	9	96062354	C	A	0.045	0.45	0.129166667	HLA-A*11:01	GQGWTVYHPT	GQGWTVYHPK	31244	128.4
RJLC3-T1	WNK2	NM_006648:exon25:c.C6137A:p.T2046K	9	96062354	C	A	0.045	0.45	0.129166667	HLA-A*11:01	QGWTVYHPT	QGWTVYHPK	33924.4	238.2
RJLC3-T1	OR10G4	NM_001004462:exon1:c.G319A:p.G107R	11	123886600	G	A	0.048	0.777777778	0.292361111	HLA-A*11:01	AQLYFFHFLG	AQLYFFHFLR	17004.3	44.2
RJLC3-T1	OR10G4	NM_001004462:exon1:c.G319A:p.G107R	11	123886600	G	A	0.048	0.777777778	0.292361111	HLA-A*11:01	QLYFFHFLG	QLYFFHFLR	15706.7	42.7
RJLC3-T1	FUT3	NM_001097641:exon2:c.A1007C:p.D336A	19	5843844	T	G	0.111	5:40	1:47	HLA-A*11:01	RSFSWALDFCK	RSFSWALAFCK	35.6	28.5
RJLC3-T1	FUT3	NM_001097641:exon2:c.A1007C:p.D336A	19	5843844	T	G	0.111	5:40	1:47	HLA-A*11:01	SFSWALDFCK	SFSWALAFCK	37.6	13.5
RJLC3-T1	FUT3	NM_001097641:exon2:c.A1007C:p.D336A	19	5843844	T	G	0.111	5:40	1:47	HLA-A*11:01	ALDFCKACWK	ALAFCKACWK	146.7	64.1
RJLC3-T1	FUT3	NM_001097641:exon2:c.A1007C:p.D336A	19	5843844	T	G	0.111	5:40	1:47	HLA-A*11:01	RSFSWALDF	RSFSWALAF	1138.2	486.1
RJLC3-T1	FUT3	NM_001097641:exon2:c.A1007C:p.D336A	19	5843844	T	G	0.111	5:40	1:47	HLA-A*11:01	FSWALDFCK	FSWALAFCK	52.7	23.7
RJLC3-T1	FUT3	NM_001097641:exon2:c.A1007C:p.D336A	19	5843844	T	G	0.111	5:40	1:47	HLA-A*11:01	LDFCKACWK	LAFCKACWK	3242.2	72.4
RJLC3-T1	PAGE2	NM_207339:exon4:c.C286G:p.P96A	X	55117857	C	G	0.029	0.456944444	0.243055556	HLA-A*11:01	IMPTFDLTK	IMATFDLTK	93.2	17.9
RJLC3-T1	ARMCX2	NM_014782:exon5:c.G1531A:p.A511T	X	100911044	C	T	0.168	32:159	0.184027778	HLA-A*11:01	LVNSIANFFR	LVNSITNFFR	57.9	48.2
RJLC3-T1	RNF17	NM_001184993:exon10:c.G1234T:p.V412L	13	25367478	G	T	0.172	36:173	0.225	HLA-B*13:01	VESSAELVF	LESSAELVF	377	258.6
RJLC3-T1	PAGE2	NM_207339:exon4:c.C286G:p.P96A	X	55117857	C	G	0.029	0.456944444	0.243055556	HLA-B*13:01	REGIMPTFDL	REGIMATFDL	799.5	490.4
RJLC3-T1	GLRA4	NM_001024452:exon7:c.C757T:p.R253W	X	102974161	G	A	0.154	26:143	0.136805556	HLA-B*13:01	LERQMGYYLI	LEWQMGYYLI	832	81.6
RJLC3-T1	GLRA4	NM_001024452:exon7:c.C757T:p.R253W	X	102974161	G	A	0.154	26:143	0.136805556	HLA-B*13:01	LERQMGYYL	LEWQMGYYL	569	47.3
RJLC3-T1	VWA5B1	NM_001039500:exon5:c.G622A:p.A208T	1	20644081	G	A	0.052	0.868055556	0.214583333	HLA-B*27:05	WNKLCLATLL	WNKLCLTTLL	508.2	461.4
RJLC3-T1	GARS	NM_002047:exon13:c.A1697C:p.Y566S	7	30665933	A	C	0.167	8:40	0.042361111	HLA-B*27:05	VKRFQKTLYV	VKRFQKTLSV	347.6	156.2
RJLC3-T1	GARS	NM_002047:exon13:c.A1697C:p.Y566S	7	30665933	A	C	0.167	8:40	0.042361111	HLA-B*27:05	KRFQKTLYVE	KRFQKTLSVE	263.9	222.9
RJLC3-T1	GARS	NM_002047:exon13:c.A1697C:p.Y566S	7	30665933	A	C	0.167	8:40	0.042361111	HLA-B*27:05	KRFQKTLYV	KRFQKTLSV	44.1	23.4
RJLC3-T1	OR10G4	NM_001004462:exon1:c.G319A:p.G107R	11	123886600	G	A	0.048	0.777777778	0.292361111	HLA-B*27:05	FLGSTECFLY	FLRSTECFLY	16946	366.9
RJLC3-T1	OR10G4	NM_001004462:exon1:c.G319A:p.G107R	11	123886600	G	A	0.048	0.777777778	0.292361111	HLA-B*27:05	LGSTECFLY	LRSTECFLY	26173.9	331.1
RJLC3-T1	TRAPPC1	NM_021210:exon4:c.C395G:p.S132C	17	7833967	G	C	0.13	0.972916667	0.115277778	HLA-B*27:05	RSRLDSYVRSL	RSRLDCYVRSL	635.8	326.4
RJLC3-T1	TRAPPC1	NM_021210:exon4:c.C395G:p.S132C	17	7833967	G	C	0.13	0.972916667	0.115277778	HLA-B*27:05	FRSRLDSYVR	FRSRLDCYVR	146.2	139.3
RJLC3-T1	TRAPPC1	NM_021210:exon4:c.C395G:p.S132C	17	7833967	G	C	0.13	0.972916667	0.115277778	HLA-B*27:05	RSRLDSYVRS	RSRLDCYVRS	870.3	397.3
RJLC3-T1	TRAPPC1	NM_021210:exon4:c.C395G:p.S132C	17	7833967	G	C	0.13	0.972916667	0.115277778	HLA-B*27:05	SRLDSYVRSL	SRLDCYVRSL	45.6	26.7
RJLC3-T1	TRAPPC1	NM_021210:exon4:c.C395G:p.S132C	17	7833967	G	C	0.13	0.972916667	0.115277778	HLA-B*27:05	FRSRLDSYV	FRSRLDCYV	551.3	493.7
RJLC3-T1	FUT3	NM_001097641:exon2:c.A1007C:p.D336A	19	5843844	T	G	0.111	5:40	1:47	HLA-B*27:05	RPRSFSWALD	RPRSFSWALA	2184	279.1
RJLC3-T1	FCRL4	NM_031282:exon7:c.G1165A:p.A389T	1	157551405	C	T	0.086	3:32	1:44	HLA-C*03:04	AAGATGGLL	TAGATGGLL	561	462.6
RJLC3-T1	LRRC66	NM_001024611:exon1:c.A355G:p.N119D	4	52883425	T	C	0.156	25:135	0.159027778	HLA-C*03:04	LSNNAIHSL	LSNDAIHSL	33.2	26.1
RJLC3-T1	PITX1	NM_002653:exon2:c.A332G:p.N111S	5	134367036	T	C	0.202	65:256	0.200694444	HLA-C*03:04	RNRYPDMSM	RSRYPDMSM	6887.4	312.8
RJLC3-T1	C2CD4A	NM_207322:exon2:c.C125T:p.T42I	15	62359937	C	T	0.149	24:137	0.113194444	HLA-C*03:04	TAACANVLT	TAACANVLI	10969.2	276.5
RJLC3-T1	CASC3	NM_007359:exon7:c.A899G:p.N300S	17	38319847	A	G	0.073	0.353472222	0.081944444	HLA-C*03:04	RNAAGTGRM	RSAAGTGRM	6128.7	165.6
RJLC3-T1	MUC16	NM_024690:exon3:c.C17771T:p.T5924I	19	9069675	G	A	0.105	0.713888889	0.134027778	HLA-C*03:04	TSSPSSPVPL	ISSPSSPVPL	1299.1	430.3
RJLC3-T1	PAGE2	NM_207339:exon4:c.C286G:p.P96A	X	55117857	C	G	0.029	0.456944444	0.243055556	HLA-C*03:04	MPTFDLTKVL	MATFDLTKVL	1228.5	186.5
RJLC3-T1	PAGE2	NM_207339:exon4:c.C286G:p.P96A	X	55117857	C	G	0.029	0.456944444	0.243055556	HLA-C*03:04	MPTFDLTKV	MATFDLTKV	3392.7	254.7
RJLC3-T1	PAGE2	NM_207339:exon4:c.C286G:p.P96A	X	55117857	C	G	0.029	0.456944444	0.243055556	HLA-C*03:04	PTFDLTKVL	ATFDLTKVL	5463.4	113.2
RJLC3-T1	VWA5B1	NM_001039500:exon5:c.G622A:p.A208T	1	20644081	G	A	0.052	0.868055556	0.214583333	HLA-C*15:02	LATLLNTEV	LTTLLNTEV	663	335.8
RJLC3-T2	NASP	NM_001195193:exon8:c.G1658C:p.R553T	1	46080868	G	C	0.186	59:258	0.207638889	HLA-A*02:01	IEVIENRMAV	IEVIENTMAV	1765.3	188
RJLC3-T2	NASP	NM_001195193:exon8:c.G1658C:p.R553T	1	46080868	G	C	0.186	59:258	0.207638889	HLA-A*02:01	NRMAVLNEQV	NTMAVLNEQV	1495.6	369.3
RJLC3-T2	PIK3CA	NM_006218:exon8:c.G1357C:p.E453Q	3	178928079	G	C	0.195	57:235	0.2125	HLA-A*02:01	GLEDLLNPIGV	GLQDLLNPIGV	688.8	147.4
RJLC3-T2	PIK3CA	NM_006218:exon8:c.G1357C:p.E453Q	3	178928079	G	C	0.195	57:235	0.2125	HLA-A*02:01	HGLEDLLNPI	HGLQDLLNPI	1026.7	246.4
RJLC3-T2	PIK3CA	NM_006218:exon8:c.G1357C:p.E453Q	3	178928079	G	C	0.195	57:235	0.2125	HLA-A*02:01	LEDLLNPIGV	LQDLLNPIGV	16812.7	489.3
RJLC3-T2	PIK3CA	NM_006218:exon8:c.G1357C:p.E453Q	3	178928079	G	C	0.195	57:235	0.2125	HLA-A*02:01	GLEDLLNPI	GLQDLLNPI	53	14.5
RJLC3-T2	SLC9B1	NM_001100874:exon6:c.A651C:p.L217F	4	103866352	T	G	0.153	0.864583333	0.088194444	HLA-A*02:01	WAFLLGFVLGA	WAFLFGFVLGA	623.6	332.9
RJLC3-T2	SLC9B1	NM_001100874:exon6:c.A651C:p.L217F	4	103866352	T	G	0.153	0.864583333	0.088194444	HLA-A*02:01	AFLLGFVLGAV	AFLFGFVLGAV	81.8	78.8
RJLC3-T2	SLC9B1	NM_001100874:exon6:c.A651C:p.L217F	4	103866352	T	G	0.153	0.864583333	0.088194444	HLA-A*02:01	FLLGFVLGAVS	FLFGFVLGAVS	191.7	189
RJLC3-T2	SLC9B1	NM_001100874:exon6:c.A651C:p.L217F	4	103866352	T	G	0.153	0.864583333	0.088194444	HLA-A*02:01	WQWAFLLGFV	WQWAFLFGFV	32.7	25
RJLC3-T2	SLC9B1	NM_001100874:exon6:c.A651C:p.L217F	4	103866352	T	G	0.153	0.864583333	0.088194444	HLA-A*02:01	AFLLGFVLGA	AFLFGFVLGA	38.1	22.5
RJLC3-T2	SLC9B1	NM_001100874:exon6:c.A651C:p.L217F	4	103866352	T	G	0.153	0.864583333	0.088194444	HLA-A*02:01	FLLGFVLGAV	FLFGFVLGAV	7.9	7.4
RJLC3-T2	SLC9B1	NM_001100874:exon6:c.A651C:p.L217F	4	103866352	T	G	0.153	0.864583333	0.088194444	HLA-A*02:01	FLLGFVLGA	FLFGFVLGA	6.6	5.1
RJLC3-T2	RIOK2	NM_001159749:exon4:c.A496C:p.K166Q	5	96512822	T	G	0.139	0.505555556	0.057638889	HLA-A*02:01	AMKEFAYMKAL	AMKEFAYMQAL	1060.2	190.1
RJLC3-T2	RIOK2	NM_001159749:exon4:c.A496C:p.K166Q	5	96512822	T	G	0.139	0.505555556	0.057638889	HLA-A*02:01	MKALYERKFPV	MQALYERKFPV	45	44.7
RJLC3-T2	MUC2	NM_002457:exon30:c.G4163T:p.C1388F	11	1092344	G	T	0.205	41:159	0.172222222	HLA-A*02:01	KIRVNCCWPM	KIRVNFCWPM	1000.6	338.8
RJLC3-T2	NASP	NM_001195193:exon8:c.G1658C:p.R553T	1	46080868	G	C	0.186	59:258	0.207638889	HLA-A*11:01	NRMAVLNEQVK	NTMAVLNEQVK	4395.8	111.9
RJLC3-T2	SRSF11	NM_001190987:exon7:c.T597G:p.N199K	1	70703114	T	G	0.162	11:57	0.047222222	HLA-A*11:01	KLMSTVDPKLN	KLMSTVDPKLK	8648.5	336.3
RJLC3-T2	SRSF11	NM_001190987:exon7:c.T597G:p.N199K	1	70703114	T	G	0.162	11:57	0.047222222	HLA-A*11:01	LMSTVDPKLN	LMSTVDPKLK	33738.4	240.6
RJLC3-T2	HDLBP	NM_005336:exon6:c.A632C:p.E211A	2	242196040	T	G	0.17	0.675694444	0.096527778	HLA-A*11:01	EVLLISAEQDK	AVLLISAEQDK	4363.8	275.1
RJLC3-T2	CXXC11	NM_173821:exon2:c.C478G:p.P160A	2	242814185	C	G	0.143	0.504166667	0.073611111	HLA-A*11:01	DPAWSANATK	DAAWSANATK	6521.6	404.9
RJLC3-T2	CXXC11	NM_173821:exon2:c.C478G:p.P160A	2	242814185	C	G	0.143	0.504166667	0.073611111	HLA-A*11:01	PAWSANATK	AAWSANATK	1625.3	40.6
RJLC3-T2	ZNF860	NM_001137674:exon2:c.C1414T:p.H472Y	3	32031985	C	T	0.217	81:293	0.261805556	HLA-A*11:01	VIHKAIHTGEK	VIYKAIHTGEK	1490.9	343.7
RJLC3-T2	ZNF860	NM_001137674:exon2:c.C1414T:p.H472Y	3	32031985	C	T	0.217	81:293	0.261805556	HLA-A*11:01	HHNSALVIHK	HHNSALVIYK	1738.7	232.8
RJLC3-T2	ZNF860	NM_001137674:exon2:c.C1414T:p.H472Y	3	32031985	C	T	0.217	81:293	0.261805556	HLA-A*11:01	HNSALVIHK	HNSALVIYK	513.9	71.1
RJLC3-T2	PIK3CA	NM_006218:exon5:c.G1054A:p.D352N	3	178921572	G	A	0.184	0.85	0.079166667	HLA-A*11:01	VNVNIRDIDK	VNVNIRDINK	1850.4	459.7
RJLC3-T2	PIK3CA	NM_006218:exon5:c.G1054A:p.D352N	3	178921572	G	A	0.184	0.85	0.079166667	HLA-A*11:01	NVNIRDIDK	NVNIRDINK	1809.3	388.4
RJLC3-T2	PIK3CA	NM_006218:exon6:c.G1093A:p.E365K	3	178922324	G	A	0.133	0.508333333	0.103472222	HLA-A*11:01	RTGIYHGGE	RTGIYHGGK	20161.3	123.1
RJLC3-T2	RIOK2	NM_001159749:exon4:c.A496C:p.K166Q	5	96512822	T	G	0.139	0.505555556	0.057638889	HLA-A*11:01	FAYMKALYERK	FAYMQALYERK	169.9	169
RJLC3-T2	RIOK2	NM_001159749:exon4:c.A496C:p.K166Q	5	96512822	T	G	0.139	0.505555556	0.057638889	HLA-A*11:01	AYMKALYERK	AYMQALYERK	508.1	311.5
RJLC3-T2	RIOK2	NM_001159749:exon4:c.A496C:p.K166Q	5	96512822	T	G	0.139	0.505555556	0.057638889	HLA-A*11:01	YMKALYERK	YMQALYERK	560	205.3
RJLC3-T2	C9orf3	NM_001193329:exon1:c.T41G:p.L14R	9	97522106	T	G	0.105	0.856944444	0.110416667	HLA-A*11:01	LMANTSHILVK	RMANTSHILVK	333.4	189.8
RJLC3-T2	MUC2	NM_002457:exon30:c.G4163T:p.C1388F	11	1092344	G	T	0.205	41:159	0.172222222	HLA-A*11:01	IRVNCCWPMDK	IRVNFCWPMDK	1157.7	407.2
RJLC3-T2	MUC2	NM_002457:exon30:c.G4163T:p.C1388F	11	1092344	G	T	0.205	41:159	0.172222222	HLA-A*11:01	RVNCCWPMDK	RVNFCWPMDK	58.6	20.8
RJLC3-T2	MUC2	NM_002457:exon30:c.G4163T:p.C1388F	11	1092344	G	T	0.205	41:159	0.172222222	HLA-A*11:01	VNCCWPMDK	VNFCWPMDK	1543.3	182.4
RJLC3-T2	SDHAF2	NM_017841:exon2:c.A218C:p.E73A	11	61205278	A	C	0.109	0.99375	0.166666667	HLA-A*11:01	RARLLYESRK	RARLLYASRK	675	188.6
RJLC3-T2	ZNF208	NM_007153:exon4:c.G1010C:p.G337A	19	22156826	C	G	0.09	0.486805556	0.092361111	HLA-A*11:01	KAIHAGEKPYK	KAIHAAEKPYK	613.9	351
RJLC3-T2	ZNF208	NM_007153:exon4:c.G1010C:p.G337A	19	22156826	C	G	0.09	0.486805556	0.092361111	HLA-A*11:01	AIHAGEKPYK	AIHAAEKPYK	117.4	77.4
RJLC3-T2	XRCC1	NM_006297:exon7:c.T670G:p.S224A	19	44057175	A	C	0.194	0.846527778	0.066666667	HLA-A*11:01	AASSASPVSR	AASAASPVSR	321.2	235.7
RJLC3-T2	XRCC1	NM_006297:exon7:c.T670G:p.S224A	19	44057175	A	C	0.194	0.846527778	0.066666667	HLA-A*11:01	ASSASPVSR	ASAASPVSR	92.7	64.6
RJLC3-T2	LILRB1	NM_001278398:exon13:c.A1643C:p.E548A	19	55147988	A	C	0.667	2:01	0:12	HLA-A*11:01	VTYAEVKHSR	VTYAAVKHSR	224.9	132
RJLC3-T2	LILRB1	NM_001278398:exon13:c.A1643C:p.E548A	19	55147988	A	C	0.667	2:01	0:12	HLA-A*11:01	QAVTYAEVK	QAVTYAAVK	1288.5	376
RJLC3-T2	SDC4	NM_002999:exon4:c.G321C:p.E107D	20	43959130	C	G	0.062	0.365277778	0.088888889	HLA-A*11:01	KLEENEVIPK	KLDENEVIPK	356.5	234.8
RJLC3-T2	SLC9B1	NM_001100874:exon6:c.A651C:p.L217F	4	103866352	T	G	0.153	0.864583333	0.088194444	HLA-B*13:01	WQWAFLLGF	WQWAFLFGF	374.1	265.7
RJLC3-T2	RIOK2	NM_001159749:exon4:c.A496C:p.K166Q	5	96512822	T	G	0.139	0.505555556	0.057638889	HLA-B*13:01	MKEFAYMKAL	MKEFAYMQAL	534.3	205.2
RJLC3-T2	RIOK2	NM_001159749:exon4:c.A496C:p.K166Q	5	96512822	T	G	0.139	0.505555556	0.057638889	HLA-B*13:01	KEFAYMKAL	KEFAYMQAL	231.4	79.7
RJLC3-T2	NASP	NM_001195193:exon8:c.G1658C:p.R553T	1	46080868	G	C	0.186	59:258	0.207638889	HLA-C*03:04	EVIENRMAV	EVIENTMAV	687	235.8
RJLC3-T2	NASP	NM_001195193:exon8:c.G1658C:p.R553T	1	46080868	G	C	0.186	59:258	0.207638889	HLA-C*03:04	VIENRMAVL	VIENTMAVL	1334.5	424.4
RJLC3-T2	TTN	NM_003319:exon45:c.A12290T:p.E4097V	2	179604581	T	A	0.034	1.10625	0.447916667	HLA-C*03:04	IIEDVDPQM	IIVDVDPQM	2380.4	370.6
RJLC3-T2	SLC9B1	NM_001100874:exon6:c.A651C:p.L217F	4	103866352	T	G	0.153	0.864583333	0.088194444	HLA-C*03:04	WAFLLGFVL	WAFLFGFVL	103	94.9
RJLC3-T2	AKR1D1	NM_001190906:exon4:c.A386T:p.D129V	7	137782619	A	T	0.286	6:15	0:37	HLA-C*03:04	MAFKPGDEI	MAFKPGVEI	14.5	7.1
RJLC3-T2	SDHAF2	NM_017841:exon2:c.A218C:p.E73A	11	61205278	A	C	0.109	0.99375	0.166666667	HLA-C*03:04	YESRKRGML	YASRKRGML	19220.7	129.8
RJLC3-T2	XRCC1	NM_006297:exon7:c.T670G:p.S224A	19	44057175	A	C	0.194	0.846527778	0.066666667	HLA-C*03:04	SAASSASPV	SAASAASPV	16.6	12.1
RJLC3-T2	HDLBP	NM_005336:exon6:c.A632C:p.E211A	2	242196040	T	G	0.17	0.675694444	0.096527778	HLA-C*15:02	KARHEVLLI	KARHAVLLI	149.5	68.5
RJLC3-T2	LIN54	NM_001288996:exon2:c.C464T:p.A155V	4	83905534	G	A	0.033	0.86875	0.267361111	HLA-C*15:02	KSGSPIVLAL	KSGSPIVLVL	285.6	271.6
RJLC3-T2	LIN54	NM_001288996:exon2:c.C464T:p.A155V	4	83905534	G	A	0.033	0.86875	0.267361111	HLA-C*15:02	KSGSPIVLA	KSGSPIVLV	1428.8	180.6
RJLC3-T2	XRCC1	NM_006297:exon7:c.T670G:p.S224A	19	44057175	A	C	0.194	0.846527778	0.066666667	HLA-C*15:02	SASPVSRAI	AASPVSRAI	214.2	198.1
RJLC3-T3	PRAMEF11	NM_001146344:exon3:c.G683A:p.R228H	1	12887174	C	T	0.061	3:46	1:41	HLA-A*02:01	FLKLRCLQKL	FLKLHCLQKL	79.3	54.4

RJLC3-T3	PRAMEF11	NM_001146344:exon3:c.G683A:p.R228H	1	12887174	C	T	0.061	3:46	1:41	HLA-A*02:01	KLRCLQKLYM	KLHCLQKLYM	2109.3	383.6
RJLC3-T3	FBXO41	NM_001080410:exon2:c.C1003T:p.R335W	2	73493713	G	A	0.208	10:38	0.04375	HLA-A*02:01	FIEELLERA	FIEELLEWA	503.7	133.6
RJLC3-T3	HRH1	NM_001098211:exon2:c.T446C:p.L149P	3	11301169	T	C	0.125	35:244	0.217361111	HLA-A*02:01	ATILGAWFL	ATIPGAWFL	257.7	61.5
RJLC3-T3	CSN1S1	NM_001025104:exon11:c.C307A:p.L103I	4	70806770	C	A	0.095	0.338194444	0:58	HLA-A*02:01	RLNEYNQLQL	RLNEYNQIQL	98.5	67.9
RJLC3-T3	PLEC	NM_201378:exon25:c.C3200A:p.S1067Y	8	145003289	G	T	0.091	4:40	0:47	HLA-A*02:01	SLSAIYLEKL	SLYAIYLEKL	104.1	49.1
RJLC3-T3	TMEM245	NM_032012:exon15:c.T2210C:p.V737A	9	111800311	A	G	0.043	0.797916667	0.272222222	HLA-A*02:01	TMFGINIVFI	TMFGINIAFI	39.1	38.6
RJLC3-T3	TMEM245	NM_032012:exon15:c.T2210C:p.V737A	9	111800311	A	G	0.043	0.797916667	0.272222222	HLA-A*02:01	GINIVFIPSA	GINIAFIPSA	1059.6	251.7
RJLC3-T3	TMEM245	NM_032012:exon15:c.T2210C:p.V737A	9	111800311	A	G	0.043	0.797916667	0.272222222	HLA-A*02:01	TMFGINIVF	TMFGINIAF	699.2	457.8
RJLC3-T3	KRAS	NM_004985:exon2:c.G34T:p.G12C	12	25398285	C	A	0.37	74:126	0.140277778	HLA-A*02:01	KLVVVGAGGV	KLVVVGACGV	392.9	203.6
RJLC3-T3	DCT	NM_001922:exon8:c.A1498G:p.K500E	13	95092214	T	C	0.08	1.041666667	0.206944444	HLA-A*02:01	RLRKGYTPL	RLREGYTPL	1051.2	138.6
RJLC3-T3	ALDH1A3	NM_000693:exon13:c.G1477A:p.A493T	15	101454916	G	A	0.036	0.725	0.252777778	HLA-A*02:01	YALAEYTEV	YTLAEYTEV	84.9	26.7
RJLC3-T3	MC2R	NM_000529:exon2:c.T184A:p.F62I	18	13885334	A	T	0.23	73:244	0.255555556	HLA-A*02:01	KNLQAPMYFF	KNLQAPMYFI	7092.3	149.4
RJLC3-T3	MC2R	NM_000529:exon2:c.T184A:p.F62I	18	13885334	A	T	0.23	73:244	0.255555556	HLA-A*02:01	NLQAPMYFF	NLQAPMYFI	3530.4	46.9
RJLC3-T3	C18orf63	NM_001174123:exon3:c.G211C:p.A71P	18	71987824	G	C	0.11	0.425694444	0.073611111	HLA-A*02:01	ILNQIWVVMAI	ILNQIWVVMPI	817.6	301.3
RJLC3-T3	C18orf63	NM_001174123:exon3:c.G211C:p.A71P	18	71987824	G	C	0.11	0.425694444	0.073611111	HLA-A*02:01	LNQIWVVMAI	LNQIWVVMPI	409.5	143.7
RJLC3-T3	C18orf63	NM_001174123:exon3:c.G211C:p.A71P	18	71987824	G	C	0.11	0.425694444	0.073611111	HLA-A*02:01	NQIWVVMAI	NQIWVVMPI	105.7	33.6
RJLC3-T3	MPP1	NM_001166460:exon1:c.C43A:p.H15N	X	154033606	G	T	0.134	0.784722222	0.110416667	HLA-A*02:01	SMHTALSDL	SMNTALSDL	964.7	471.4
RJLC3-T3	FSTL1	NM_007085:exon10:c.G806A:p.G269E	3	120118806	C	T	0.127	0.743055556	0.099305556	HLA-A*11:01	VCTAMTCDGK	VCTAMTCDEK	306.4	258.2
RJLC3-T3	PCDHB13	NM_018933:exon1:c.C889T:p.P297S	5	140594584	C	T	0.082	26:292	0.2375	HLA-A*11:01	NPLTGEIELK	NSLTGEIELK	9636.1	264
RJLC3-T3	PCDHB13	NM_018933:exon1:c.C889T:p.P297S	5	140594584	C	T	0.082	26:292	0.2375	HLA-A*11:01	PLTGEIELKK	SLTGEIELKK	2531.3	173.7
RJLC3-T3	PCDHB13	NM_018933:exon1:c.C889T:p.P297S	5	140594584	C	T	0.082	26:292	0.2375	HLA-A*11:01	PLTGEIELK	SLTGEIELK	10884.2	272.1
RJLC3-T3	PLEC	NM_201378:exon25:c.C3200A:p.S1067Y	8	145003289	G	T	0.091	4:40	0:47	HLA-A*11:01	RSLSAIYLEK	RSLYAIYLEK	15.9	14.8
RJLC3-T3	PLEC	NM_201378:exon25:c.C3200A:p.S1067Y	8	145003289	G	T	0.091	4:40	0:47	HLA-A*11:01	SLSAIYLEK	SLYAIYLEK	8.2	7.6
RJLC3-T3	KRAS	NM_004985:exon2:c.G34T:p.G12C	12	25398285	C	A	0.37	74:126	0.140277778	HLA-A*11:01	VVVGAGGVGK	VVVGACGVGK	271.4	165.8
RJLC3-T3	KRAS	NM_004985:exon2:c.G34T:p.G12C	12	25398285	C	A	0.37	74:126	0.140277778	HLA-A*11:01	VVGAGGVGK	VVGACGVGK	139.3	90.6
RJLC3-T3	ALDH1A3	NM_000693:exon13:c.G1477A:p.A493T	15	101454916	G	A	0.036	0.725	0.252777778	HLA-A*11:01	YALAEYTEVK	YTLAEYTEVK	416.4	74.3
RJLC3-T3	C18orf63	NM_001174123:exon3:c.G211C:p.A71P	18	71987824	G	C	0.11	0.425694444	0.073611111	HLA-A*11:01	IWVVMAIPFYK	IWVVMPIPFYK	11.1	10.1
RJLC3-T3	C18orf63	NM_001174123:exon3:c.G211C:p.A71P	18	71987824	G	C	0.11	0.425694444	0.073611111	HLA-A*11:01	WVVMAIPFYKA	WVVMPIPFYKA	197.1	152.6
RJLC3-T3	C18orf63	NM_001174123:exon3:c.G211C:p.A71P	18	71987824	G	C	0.11	0.425694444	0.073611111	HLA-A*11:01	WVVMAIPFYK	WVVMPIPFYK	6.2	5.8
RJLC3-T3	FUT3	NM_001097641:exon2:c.A1007C:p.D336A	19	5843844	T	G	0.06	3:47	1:47	HLA-A*11:01	RSFSWALDFCK	RSFSWALAFCK	35.6	28.5
RJLC3-T3	FUT3	NM_001097641:exon2:c.A1007C:p.D336A	19	5843844	T	G	0.06	3:47	1:47	HLA-A*11:01	SFSWALDFCK	SFSWALAFCK	37.6	13.5
RJLC3-T3	FUT3	NM_001097641:exon2:c.A1007C:p.D336A	19	5843844	T	G	0.06	3:47	1:47	HLA-A*11:01	ALDFCKACWK	ALAFCKACWK	146.7	64.1
RJLC3-T3	FUT3	NM_001097641:exon2:c.A1007C:p.D336A	19	5843844	T	G	0.06	3:47	1:47	HLA-A*11:01	RSFSWALDF	RSFSWALAF	1138.2	486.1
RJLC3-T3	FUT3	NM_001097641:exon2:c.A1007C:p.D336A	19	5843844	T	G	0.06	3:47	1:47	HLA-A*11:01	FSWALDFCK	FSWALAFCK	52.7	23.7
RJLC3-T3	FUT3	NM_001097641:exon2:c.A1007C:p.D336A	19	5843844	T	G	0.06	3:47	1:47	HLA-A*11:01	LDFCKACWK	LAFCKACWK	3242.2	72.4
RJLC3-T3	MPP1	NM_001166460:exon1:c.C43A:p.H15N	X	154033606	G	T	0.134	0.784722222	0.110416667	HLA-A*11:01	GSMHTALSDLY	GSMNTALSDLY	370.9	314.3
RJLC3-T3	MPP1	NM_001166460:exon1:c.C43A:p.H15N	X	154033606	G	T	0.134	0.784722222	0.110416667	HLA-A*11:01	SMHTALSDLY	SMNTALSDLY	683.2	432.8
RJLC3-T3	LAMB2	NM_002292:exon17:c.C2281T:p.P761S	3	49163463	G	A	0.15	30:170	0.135416667	HLA-B*13:01	PSEACAPLLI	SSEACAPLLI	1142.7	328.5
RJLC3-T3	PLEC	NM_201378:exon25:c.C3200A:p.S1067Y	8	145003289	G	T	0.091	4:40	0:47	HLA-B*13:01	LEQVRSLSAI	LEQVRSLYAI	953	317.6
RJLC3-T3	PLEC	NM_201378:exon25:c.C3200A:p.S1067Y	8	145003289	G	T	0.091	4:40	0:47	HLA-B*13:01	EQVRSLSAI	EQVRSLYAI	1165.5	194
RJLC3-T3	LAMB2	NM_002292:exon17:c.C2281T:p.P761S	3	49163463	G	A	0.15	30:170	0.135416667	HLA-C*03:04	SPSEACAPL	SSSEACAPL	638.8	33.9
RJLC3-T3	TMEM245	NM_032012:exon15:c.T2210C:p.V737A	9	111800311	A	G	0.043	0.797916667	0.272222222	HLA-C*03:04	NIVFIPSAL	NIAFIPSAL	671	419.2
RJLC3-T3	TMEM245	NM_032012:exon15:c.T2210C:p.V737A	9	111800311	A	G	0.043	0.797916667	0.272222222	HLA-C*03:04	IVFIPSAL	IAFIPSAL	2381.1	484.5
RJLC4-T1	RSG1	NM_030907:exon4:c.G505A:p.V169I	1	16559027	C	T	0.122	0.420138889	0.046527778	HLA-A*11:01	GVVRMVIGSK	GVIRMVIGSK	81.5	46
RJLC4-T1	SRSF11	NM_001190987:exon7:c.T597G:p.N199K	1	70703114	T	G	0.194	12:50	0.04375	HLA-A*11:01	KLMSTVDPKLN	KLMSTVDPKLK	8648.5	336.3
RJLC4-T1	SRSF11	NM_001190987:exon7:c.T597G:p.N199K	1	70703114	T	G	0.194	12:50	0.04375	HLA-A*11:01	LMSTVDPKLN	LMSTVDPKLK	33738.4	240.6
RJLC4-T1	LRIG2	NM_014813:exon12:c.C1421G:p.P474R	1	113650323	C	G	0.216	61:222	0.145138889	HLA-A*11:01	SVNVSCAHP	SVNVSCAHR	28463.9	363
RJLC4-T1	NBPF8	NM_001037501:exon12:c.T1721G:p.M574R	1	148017582	A	C	0.25	3:09	1:13	HLA-A*11:01	STLSIPPEM	STLSIPPER	6287.6	80.1
RJLC4-T1	SULT1C3	NM_001008743:exon1:c.C29T:p.T10M	2	108863679	C	T	0.152	48:267	0.281944444	HLA-A*11:01	KIEKNAPTMEK	KIEKNAPMMEK	134	118.2
RJLC4-T1	ZFP62	NM_001172638:exon2:c.T2399G:p.L800R	5	180276096	A	C	0.328	79:162	0.154861111	HLA-A*11:01	KAYISHSSL	KAYISHSSR	22978.2	256.4
RJLC4-T1	RP1-257A7.4	NM_001242698:exon4:c.G186C:p.M62I	6	13281740	C	G	0.19	0.713888889	0.045138889	HLA-A*11:01	AMISAHCNLR	AIISAHCNLR	415.6	254.1
RJLC4-T1	VPS13B	NM_017890:exon48:c.A8849C:p.N2950T	8	100831792	A	C	0.08	0.645833333	0.122222222	HLA-A*11:01	SLQPIWPYNKK	SLQPIWPYTKK	148.3	141.5
RJLC4-T1	VPS13B	NM_017890:exon48:c.A8849C:p.N2950T	8	100831792	A	C	0.08	0.645833333	0.122222222	HLA-A*11:01	SLQPIWPYNK	SLQPIWPYTK	25.7	25.3
RJLC4-T1	VPS13B	NM_017890:exon48:c.A8849C:p.N2950T	8	100831792	A	C	0.08	0.645833333	0.122222222	HLA-A*11:01	LQPIWPYNKK	LQPIWPYTKK	494.4	319.9
RJLC4-T1	HHLA1	NM_001145095:exon8:c.C561G:p.I187M	8	133100120	G	C	0.341	72:139	0.152777778	HLA-A*11:01	IFICVMTGK	MFICVMTGK	701.1	317.4
RJLC4-T1	C9orf3	NM_001193329:exon1:c.T41G:p.L14R	9	97522106	T	G	0.108	0.852777778	0.127777778	HLA-A*11:01	LMANTSHILVK	RMANTSHILVK	333.4	189.8
RJLC4-T1	COQ4	NM_016035:exon2:c.A188G:p.N63S	9	131085412	A	G	0.123	0.652777778	0.045138889	HLA-A*11:01	SAAMALYNPYR	SAAMALYSPYR	495.7	396.9
RJLC4-T1	COQ4	NM_016035:exon2:c.A188G:p.N63S	9	131085412	A	G	0.123	0.652777778	0.045138889	HLA-A*11:01	SAAMALYNPY	SAAMALYSPY	242.3	161.6
RJLC4-T1	COQ4	NM_016035:exon2:c.A188G:p.N63S	9	131085412	A	G	0.123	0.652777778	0.045138889	HLA-A*11:01	AAMALYNPYR	AAMALYSPYR	36.9	25.2
RJLC4-T1	COQ4	NM_016035:exon2:c.A188G:p.N63S	9	131085412	A	G	0.123	0.652777778	0.045138889	HLA-A*11:01	AAMALYNPY	AAMALYSPY	78.1	49.4
RJLC4-T1	COQ4	NM_016035:exon2:c.A188G:p.N63S	9	131085412	A	G	0.123	0.652777778	0.045138889	HLA-A*11:01	AMALYNPYR	AMALYSPYR	83	54.9
RJLC4-T1	MED27	NM_004269:exon5:c.G607T:p.V203F	9	134769346	C	A	0.1	0.622916667	0.095833333	HLA-A*11:01	IVVMRSLFIDR	IVFMRSLFIDR	1601	314.8
RJLC4-T1	MED27	NM_004269:exon5:c.G607T:p.V203F	9	134769346	C	A	0.1	0.622916667	0.095833333	HLA-A*11:01	KVLKVIVVMR	KVLKVIVFMR	661.6	186.3
RJLC4-T1	SCUBE2	NM_001170690:exon18:c.G2411C:p.R804T	11	9042605	C	G	0.148	31:178	0.176388889	HLA-A*11:01	SKVSRFLRPYK	SKVSRFLTPYK	198	97.8
RJLC4-T1	SCUBE2	NM_001170690:exon18:c.G2411C:p.R804T	11	9042605	C	G	0.148	31:178	0.176388889	HLA-A*11:01	SKVSRFLRPY	SKVSRFLTPY	292.5	106.8
RJLC4-T1	SCUBE2	NM_001170690:exon18:c.G2411C:p.R804T	11	9042605	C	G	0.148	31:178	0.176388889	HLA-A*11:01	KVSRFLRPYK	KVSRFLTPYK	11.7	8.7
RJLC4-T1	SCUBE2	NM_001170690:exon18:c.G2411C:p.R804T	11	9042605	C	G	0.148	31:178	0.176388889	HLA-A*11:01	KVSRFLRPY	KVSRFLTPY	136.9	50.5
RJLC4-T1	SCUBE2	NM_001170690:exon18:c.G2411C:p.R804T	11	9042605	C	G	0.148	31:178	0.176388889	HLA-A*11:01	VSRFLRPYK	VSRFLTPYK	115.9	33.3
RJLC4-T1	GPRC5A	NM_003979:exon2:c.T133A:p.F45I	12	13061316	T	A	0.24	56:177	0.284027778	HLA-A*11:01	FMLTLPILVCK	IMLTLPILVCK	222.3	151.7
RJLC4-T1	LDHD	NM_153486:exon2:c.T133G:p.S45A	16	75149498	A	C	0.165	0.813194444	0.058333333	HLA-A*11:01	SHVSTAAVVR	SHVATAAVVR	570.7	496.5
RJLC4-T1	TP53	NM_001126115:exon3:c.G350A:p.R117K	17	7577535	C	T	0.264	19:53	0.06875	HLA-A*11:01	NSSCMGGMNRR	NSSCMGGMNRK	2684.2	307.2
RJLC4-T1	TP53	NM_001126115:exon3:c.G350A:p.R117K	17	7577535	C	T	0.264	19:53	0.06875	HLA-A*11:01	SSCMGGMNRR	SSCMGGMNRK	114.2	14.6
RJLC4-T1	TP53	NM_001126115:exon3:c.G350A:p.R117K	17	7577535	C	T	0.264	19:53	0.06875	HLA-A*11:01	SCMGGMNRR	SCMGGMNRK	4430.2	472.7
RJLC4-T1	MAP3K3	NM_002401:exon6:c.G422A:p.R141Q	17	61744340	G	A	0.384	83:133	0.136805556	HLA-A*11:01	SGVSRQVRIK	SGVSRQVQIK	530.3	282.1
RJLC4-T1	MAP3K3	NM_002401:exon6:c.G422A:p.R141Q	17	61744340	G	A	0.384	83:133	0.136805556	HLA-A*11:01	GVSRQVRIK	GVSRQVQIK	368	159.6
RJLC4-T1	TXNL4A	NM_006701:exon2:c.G241A:p.V81I	18	77737614	C	T	0.174	1.034027778	0.097222222	HLA-A*11:01	CTVMFFFRNK	CTIMFFFRNK	19.2	18.9
RJLC4-T1	FBXL12	NM_017703:exon1:c.C76T:p.R26C	19	9929414	G	A	0.172	35:169	0.140972222	HLA-A*11:01	RIRISRVCHR	RICISRVCHR	464.1	416.3
RJLC4-T1	S1PR5	NM_001166215:exon2:c.A298G:p.T100A	19	10625390	T	C	0.151	34:191	0.2125	HLA-A*11:01	NILLSGPLTLK	NILLSGPLALK	438.1	409.3
RJLC4-T1	S1PR5	NM_001166215:exon2:c.A298G:p.T100A	19	10625390	T	C	0.151	34:191	0.2125	HLA-A*11:01	LLSGPLTLK	LLSGPLALK	33	32.5
RJLC4-T1	MAP3K10	NM_002446:exon9:c.C2270T:p.S757F	19	40719856	C	T	0.324	55:115	0.090277778	HLA-A*11:01	SSVSDCNSTR	SSVSDCNFTR	636.7	170.8
RJLC4-T1	MAP3K10	NM_002446:exon9:c.C2270T:p.S757F	19	40719856	C	T	0.324	55:115	0.090277778	HLA-A*11:01	SVSDCNSTR	SVSDCNFTR	222.1	58.3
RJLC4-T1	LRIG2	NM_014813:exon12:c.C1421G:p.P474R	1	113650323	C	G	0.216	61:222	0.145138889	HLA-B*40:01	HPEWLAGQSIL	HREWLAGQSIL	3413.9	432.7
RJLC4-T1	LRIG2	NM_014813:exon12:c.C1421G:p.P474R	1	113650323	C	G	0.216	61:222	0.145138889	HLA-B*40:01	HPEWLAGQSI	HREWLAGQSI	78.7	75.3
RJLC4-T1	LRIG2	NM_014813:exon12:c.C1421G:p.P474R	1	113650323	C	G	0.216	61:222	0.145138889	HLA-B*40:01	PEWLAGQSIL	REWLAGQSIL	1544	19.7
RJLC4-T1	LRIG2	NM_014813:exon12:c.C1421G:p.P474R	1	113650323	C	G	0.216	61:222	0.145138889	HLA-B*40:01	PEWLAGQSI	REWLAGQSI	512.7	9.1
RJLC4-T1	IGSF8	NM_052868:exon5:c.G1474A:p.V492I	1	160062324	C	T	0.075	0.351388889	0.059722222	HLA-B*40:01	GELSSVPAQL	GELSSIPAQL	34.8	33.5
RJLC4-T1	RP1-257A7.4	NM_001242698:exon4:c.G186C:p.M62I	6	13281740	C	G	0.19	0.713888889	0.045138889	HLA-B*40:01	LEFNGAMI	LEFNGAII	350.1	184.7
RJLC4-T1	TONSL	NM_013432:exon15:c.C1852T:p.L618F	8	145662178	G	A	0.187	34:148	0.136805556	HLA-B*40:01	LLERGASVTL	LFERGASVTL	176.8	152.6
RJLC4-T1	TONSL	NM_013432:exon15:c.C1852T:p.L618F	8	145662178	G	A	0.187	34:148	0.136805556	HLA-B*40:01	LERGASVTL	FERGASVTL	23.2	14.4
RJLC4-T1	TMC3	NM_001080532:exon14:c.C1535A:p.S512Y	15	81636370	G	T	0.193	37:155	0.101388889	HLA-B*40:01	QEMLKLSII	QEMLKLYII	147.4	107.6
RJLC4-T1	TXNL4A	NM_006701:exon2:c.G241A:p.V81I	18	77737614	C	T	0.174	1.034027778	0.097222222	HLA-B*40:01	YELYDPCTV	YELYDPCTI	113.4	41.3
RJLC4-T1	IL2RB	NM_000878:exon6:c.T481C:p.F161L	22	37533683	A	G	0.164	0.904166667	0.150694444	HLA-B*40:01	WEISQASHYF	WEISQASHYL	339.2	25.1
RJLC4-T1	LONRF3	NM_024778:exon6:c.A1505C:p.E502A	X	118143186	A	C	0.102	0.621527778	0.133333333	HLA-B*40:01	EEEMEELSNL	AEEMEELSNL	1738.3	107
RJLC4-T1	IGSF8	NM_052868:exon5:c.G1474A:p.V492I	1	160062324	C	T	0.075	0.351388889	0.059722222	HLA-B*55:02	VPAQLVGGV	IPAQLVGGV	301.8	193.8
RJLC4-T1	PCDHA2	NM_018905:exon1:c.C1867T:p.R623C	5	140176416	C	T	0.185	46:202	0.155555556	HLA-B*55:02	RIPFRVGLYT	RIPFCVGLYT	635.5	293.6
RJLC4-T1	PCDHA2	NM_018905:exon1:c.C1867T:p.R623C	5	140176416	C	T	0.185	46:202	0.155555556	HLA-B*55:02	IPFRVGLYTG	IPFCVGLYTG	381.3	193.6
RJLC4-T1	PCDHA2	NM_018905:exon1:c.C1867T:p.R623C	5	140176416	C	T	0.185	46:202	0.155555556	HLA-B*55:02	IPFRVGLYT	IPFCVGLYT	78.2	59.3
RJLC4-T1	RP1-257A7.4	NM_001242698:exon4:c.G186C:p.M62I	6	13281740	C	G	0.19	0.713888889	0.045138889	HLA-B*55:02	LPRLEFNGAM	LPRLEFNGAI	437.8	199.3
RJLC4-T1	C4B	NM_001002029:exon28:c.G3629T:p.R1210L	6	31997068	G	T	0.072	0.505555556	0.099305556	HLA-B*55:02	KAPADLRGVA	KAPADLLGVA	829.2	468.9
RJLC4-T1	C4B	NM_001002029:exon28:c.G3629T:p.R1210L	6	31997068	G	T	0.072	0.505555556	0.099305556	HLA-B*55:02	APADLRGVA	APADLLGVA	147.6	62.8
RJLC4-T1	ICK	NM_014920:exon6:c.C460A:p.P154T	6	52884056	G	T	0.03	0.513888889	0.214583333	HLA-B*55:02	PPYTDYVST	TPYTDYVST	10133.4	277.4
RJLC4-T1	COQ4	NM_016035:exon2:c.A188G:p.N63S	9	131085412	A	G	0.123	0.652777778	0.045138889	HLA-B*55:02	NPYRHDMVAV	SPYRHDMVAV	220.5	120.2
RJLC4-T1	COQ4	NM_016035:exon2:c.A188G:p.N63S	9	131085412	A	G	0.123	0.652777778	0.045138889	HLA-B*55:02	NPYRHDMVA	SPYRHDMVA	59.6	36.3
RJLC4-T1	THOC1	NM_005131:exon1:c.C11A:p.T4K	18	268009	G	T	0.312	65:143	0.138888889	HLA-B*55:02	MSPTPPLFSL	MSPKPPLFSL	161.9	118.1
RJLC4-T1	THOC1	NM_005131:exon1:c.C11A:p.T4K	18	268009	G	T	0.312	65:143	0.138888889	HLA-B*55:02	SPTPPLFSL	SPKPPLFSL	418.3	273.3
RJLC4-T1	FBXL12	NM_017703:exon1:c.C76T:p.R26C	19	9929414	G	A	0.172	35:169	0.140972222	HLA-B*55:02	LPVRDRIRI	LPVRDRICI	415.1	371.7
RJLC4-T1	S1PR5	NM_001166215:exon2:c.A298G:p.T100A	19	10625390	T	C	0.151	34:191	0.2125	HLA-B*55:02	GPLTLKLSPA	GPLALKLSPA	411.1	256.3
RJLC4-T1	RSG1	NM_030907:exon4:c.G505A:p.V169I	1	16559027	C	T	0.122	0.420138889	0.046527778	HLA-C*03:04	IAGEAPGVV	IAGEAPGVI	333.5	221.4
RJLC4-T1	NFASC	NM_001005388:exon25:c.C2972T:p.T991M	1	204966487	C	T	0.129	26:175	0.115972222	HLA-C*03:04	TTAAATTTT	TTAAATTTM	4991.4	24.1
RJLC4-T1	SULT1C3	NM_001008743:exon1:c.C29T:p.T10M	2	108863679	C	T	0.152	48:267	0.281944444	HLA-C*03:04	MAKIEKNAPT	MAKIEKNAPM	11231.1	76.8
RJLC4-T1	SLC9B1	NM_001100874:exon6:c.A651C:p.L217F	4	103866352	T	G	0.118	0.702777778	0.127083333	HLA-C*03:04	WAFLLGFVL	WAFLFGFVL	103	94.9
RJLC4-T1	IRX1	NM_024337:exon2:c.C1007T:p.P336L	5	3600069	C	T	0.054	0.859722222	0.125694444	HLA-C*03:04	GAPKASPPP	GAPKASPPL	33700.9	414.1
RJLC4-T1	RP1-257A7.4	NM_001242698:exon4:c.G186C:p.M62I	6	13281740	C	G	0.19	0.713888889	0.045138889	HLA-C*03:04	AMISAHCNL	AIISAHCNL	310.4	250
RJLC4-T1	C4B	NM_001002029:exon28:c.G3629T:p.R1210L	6	31997068	G	T	0.072	0.505555556	0.099305556	HLA-C*03:04	RGVAHNNLM	LGVAHNNLM	579.6	256.4
RJLC4-T1	TONSL	NM_013432:exon15:c.C1852T:p.L618F	8	145662178	G	A	0.187	34:148	0.136805556	HLA-C*03:04	LLLERGASV	LLFERGASV	1061.3	356.1

RJLC4-T1	MED27	NM_004269:exon5:c.G607T:p.V203F	9	134769346	C	A	0.1	0.622916667	0.095833333	HLA-C*03:04	IVVMRSLFI	IVFMRSLFI	1310.5	488.4
RJLC4-T1	GPRC5A	NM_003979:exon2:c.T133A:p.F45I	12	13061316	T	A	0.24	56:177	0.284027778	HLA-C*03:04	VAFMLTLPI	VAIMLTLPI	15.6	13.5
RJLC4-T1	TMC3	NM_001080532:exon14:c.C1535A:p.S512Y	15	81636370	G	T	0.193	37:155	0.101388889	HLA-C*03:04	SIIDMLFTV	YIIDMLFTV	886.7	156
RJLC4-T1	S1PR5	NM_001166215:exon2:c.A298G:p.T100A	19	10625390	T	C	0.151	34:191	0.2125	HLA-C*03:04	ILLSGPLTL	ILLSGPLAL	89.9	52.6
RJLC4-T1	S1PR5	NM_001166215:exon2:c.A298G:p.T100A	19	10625390	T	C	0.151	34:191	0.2125	HLA-C*03:04	LTLKLSPAL	LALKLSPAL	71.5	15.9
RJLC4-T2	SRSF11	NM_001190987:exon7:c.T597G:p.N199K	1	70703114	T	G	0.141	9:55	0.04375	HLA-A*11:01	KLMSTVDPKLN	KLMSTVDPKLK	8648.5	336.3
RJLC4-T2	SRSF11	NM_001190987:exon7:c.T597G:p.N199K	1	70703114	T	G	0.141	9:55	0.04375	HLA-A*11:01	LMSTVDPKLN	LMSTVDPKLK	33738.4	240.6
RJLC4-T2	ADCY10	NM_018417:exon4:c.G261T:p.L87F	1	167871285	C	A	0.145	9:53	0.102083333	HLA-A*11:01	KVLIFGGDILK	KVFIFGGDILK	201.3	116.6
RJLC4-T2	CCDC132	NM_017667:exon25:c.A2460C:p.L820F	7	92979342	A	C	0.076	0.551388889	0.132638889	HLA-A*11:01	QHNIYVDALLK	QHNIYVDALFK	810.7	375.5
RJLC4-T2	CCDC132	NM_017667:exon25:c.A2460C:p.L820F	7	92979342	A	C	0.076	0.551388889	0.132638889	HLA-A*11:01	HNIYVDALLK	HNIYVDALFK	73	32.4
RJLC4-T2	CCDC132	NM_017667:exon25:c.A2460C:p.L820F	7	92979342	A	C	0.076	0.551388889	0.132638889	HLA-A*11:01	NIYVDALLK	NIYVDALFK	51.9	24.4
RJLC4-T2	MYOF	NM_133337:exon34:c.A3833C:p.Y1278S	10	95111003	T	G	0.085	0.736805556	0.139583333	HLA-A*11:01	LYMVPQGIR	LSMVPQGIR	9937.8	479.3
RJLC4-T2	UBL7	NM_001286740:exon5:c.A397G:p.M133V	15	74743852	T	C	0.034	0.36875	0.144444444	HLA-A*11:01	AVFKMLSNKE	AVFKVLSNKE	224.9	221.9
RJLC4-T2	KCNQ5	NM_001160134:exon10:c.T1260G:p.F420L	6	73900308	T	G	0.115	0.422916667	0.067361111	HLA-B*40:01	GEASSRIMKF	GEASSRIMKL	508.4	93.2
RJLC4-T2	C5	NM_001735:exon8:c.T765G:p.F255L	9	123789546	A	C	0.111	0.425	0.055555556	HLA-B*40:01	FEITIKARYF	FEITIKARYL	1198.4	112.4
RJLC4-T2	UBL7	NM_001286740:exon5:c.A397G:p.M133V	15	74743852	T	C	0.034	0.36875	0.144444444	HLA-B*40:01	REAVFKML	REAVFKVL	720.9	326.3
RJLC4-T2	LMO7	NM_015842:exon25:c.A3671T:p.E1224V	13	76427233	A	T	0.055	0.804166667	0.224305556	HLA-B*55:02	LPGIMRRGE	LPGIMRRGV	9281.9	355.4
RJLC4-T2	NAA11	NM_032693:exon1:c.A188T:p.E63V	4	80246844	T	A	0.035	0.605555556	0.220833333	HLA-C*03:04	YVLAKMEEE	YVLAKMEEV	18273.4	248
RJLC4-T2	SLC9B1	NM_001100874:exon6:c.A651C:p.L217F	4	103866352	T	G	0.168	0.811805556	0.127083333	HLA-C*03:04	WAFLLGFVL	WAFLFGFVL	103	94.9
RJLC4-T2	CCDC132	NM_017667:exon25:c.A2460C:p.L820F	7	92979342	A	C	0.076	0.551388889	0.132638889	HLA-C*03:04	YVDALLKEF	YVDALFKEF	383.6	227.9
RJLC4-T2	PAK6	NM_001276717:exon3:c.C224T:p.A75V	15	40558062	C	T	0.066	0.669444444	0.151388889	HLA-C*03:04	VVRGSAMPV	VVRGSVMPV	435	419.9
RJLC4-T3	MROH7	NM_001039464:exon19:c.C3214T:p.R1072W	1	55166924	C	T	0.103	0.381944444	0.063888889	HLA-A*11:01	KAVFKGRDQK	KAVFKGWDQK	243.9	129.5
RJLC4-T3	MROH7	NM_001039464:exon19:c.C3214T:p.R1072W	1	55166924	C	T	0.103	0.381944444	0.063888889	HLA-A*11:01	AVFKGRDQK	AVFKGWDQK	23.3	15.7
RJLC4-T3	SRSF11	NM_001190987:exon7:c.T597G:p.N199K	1	70703114	T	G	0.167	8:40	0.04375	HLA-A*11:01	KLMSTVDPKLN	KLMSTVDPKLK	8648.5	336.3
RJLC4-T3	SRSF11	NM_001190987:exon7:c.T597G:p.N199K	1	70703114	T	G	0.167	8:40	0.04375	HLA-A*11:01	LMSTVDPKLN	LMSTVDPKLK	33738.4	240.6
RJLC4-T3	HSD3B1	NM_000862:exon4:c.C689T:p.A230V	1	120056835	C	T	0.042	0.402083333	0.114583333	HLA-A*11:01	VAWAHILALR	VVWAHILALR	322.8	204.6
RJLC4-T3	MRS2	NM_001286266:exon3:c.A266T:p.Y89F	6	24412451	A	T	0.296	8:19	1:28	HLA-A*11:01	SVAPVFTVYLK	SVAPVFTVFLK	17.2	13.2
RJLC4-T3	MRS2	NM_001286266:exon3:c.A266T:p.Y89F	6	24412451	A	T	0.296	8:19	1:28	HLA-A*11:01	VAPVFTVYLK	VAPVFTVFLK	55.6	37.9
RJLC4-T3	ALDH1A3	NM_000693:exon9:c.C997T:p.R333W	15	101440893	C	T	0.05	0.439583333	0.120833333	HLA-A*11:01	FVRRSVEYAK	FVRWSVEYAK	267.8	127.3
RJLC4-T3	MRS2	NM_001286266:exon3:c.A266T:p.Y89F	6	24412451	A	T	0.296	8:19	1:28	HLA-B*55:02	APVFTVYLKA	APVFTVFLKA	203.9	112.7
RJLC4-T3	TTC21B	NM_024753:exon18:c.A2456C:p.E819A	2	166767842	T	G	0.128	0.463888889	0.109722222	HLA-C*03:04	LAHEPVNEL	LAHAPVNEL	11.9	10.2
RJLC4-T3	MUC4	NM_018406:exon2:c.C6506T:p.A2169V	3	195511945	G	A	0.333	2:04	0:10	HLA-C*03:04	ASTGHATSL	VSTGHATSL	51.7	25.6
RJLC4-T3	SLC9B1	NM_001100874:exon6:c.A651C:p.L217F	4	103866352	T	G	0.167	0.8125	0.127083333	HLA-C*03:04	WAFLLGFVL	WAFLFGFVL	103	94.9
RJLC4-T3	MRS2	NM_001286266:exon3:c.A266T:p.Y89F	6	24412451	A	T	0.296	8:19	1:28	HLA-C*03:04	SVAPVFTVY	SVAPVFTVF	757.8	47.8
RJLC4-T3	EML3	NM_153265:exon19:c.A2246C:p.E749A	11	62371212	T	G	0.093	0.532638889	0.060416667	HLA-C*03:04	MSNSGDYEI	MSNSGDYAI	150	53.8
RJLC4-T3	MGA	NM_001164273:exon15:c.A5167C:p.I1723L	15	42035325	A	C	0.096	0.675	0.13125	HLA-C*03:04	SSLGSVPII	SSLGSVPIL	246.1	75.9

Supplementary Table 5: Clonal numbers inferred by PyClone or SciClone analyses

Sample ID	PyClone	SciClone
RJLC1-T1R1	2	1
RJLC1-T1R2	3	3
RJLC1-T1R3	2	2
RJLC1-T1R4	2	3
RJLC1-T2	2	3
RJLC2-T1	2	1
RJLC2-T2	2	1
RJLC2-T3	2	2
RJLC3-T1	4	5
RJLC3-T2	2	6
RJLC3-T3	4	4
RJLC4-T1R1	5	4
RJLC4-T1R2	4	4
RJLC4-T1R3	5	3
RJLC4-T2	2	1
RJLC4-T3	3	6

Supplementary Table 6: Branch:trunk indices (ITB) from WES for MSLCs

Patient ID	Age	Sex	TNM stage	Smoking status	branch/trunk	ITB
RJLC1-T1	64	Female	pTisN0M0	Never	30/23	1.304
RJLC4-T1	60	Female	pT1N0M0	Never	60/35	1.714
L001	59	Female	pT2aN1M0	Former	4/15	0.267
L002	78	Male	pT3N2M0	Current	16/12	1.333
L004	73	Male	pT3N0M0	Current	7/23	0.304
L011	49	Female	pT2aN0M0	Current	3/29	0.103
LS01	69	Male	pT2aN0M0	Former	3/24	0.125
317	55	Male	N0	Former	16/1424	0.011
499	73	Female	N0	Former	2/98	0.02
292	57	Female	N0	Never	2/20	0.1
324	68	Female	N0	Current	23/147	0.156
283	50	Male	N2	Former	2/11	0.182
339	73	Male	N0	Never	13/40	0.325
472	74	Female	N0	Former	16/41	0.39
330	59	Female	N0	Former	116/223	0.52
4990	75	Male	N1	Former	107/138	0.775
270	62	Female	N1	Former	71/72	0.986
356	69	Female	N0	Never	27/27	1

Supplementary Table 7: Mutation co-occurrence in the same cancer gene of MSLC

	Pa1		Pa4		Pa5		Pa6			L003	
	**T1**	**T3**	**T1**	**T2**	**T1**	**T2**	**T1**	**T2**	**T3**	**R2**	**R4**
**EGFR**			L858R	S229C	L858R	L858R	L858R	L858R	L858R	L858R	L858R
**KRAS**	G12A	G12V									

Pa1,4,5,6 (Liu, Y. et al. Genomic heterogeneity of multiple synchronous lung cancer. *Nat Comun* 7, 13200 (2016))
L003 (de Bruin, E.C. et al. Spatial and temporal diversity in genomic instability processes defines lung cancer evolution. *Science* 346, 251-256 (2014))
